# Spatially explicit action research for coastal fisheries management

**DOI:** 10.1371/journal.pone.0199841

**Published:** 2018-07-11

**Authors:** Tara N. Lawrence, R. S. Bhalla

**Affiliations:** Foundation for Ecological Research, Advocacy and Learning, Morattandi, Auroville Post, Tamil Nadu, India; University of Minnesota, UNITED STATES

## Abstract

We worked with artisanal fisherfolk along the Coromandel coast in two districts of Tamil Nadu and the Union Territory of Pondicherry in South India to map and quantify catch, gear and crew details for all fishing craft along 120 km. Spatially explicit fisheries data were collected to understand the distribution of fishing effort and to identify high pressure fishing zones. Approximately 7,945 square kilometres of fishing grounds were surveyed and 3,427 fishing trips were observed using nine GPS enabled echo-sounders operated by fishermen. Data were visualised and non parametric statistical analysis revealed distinct patterns in fishing effort, high density fishing zones and large overlaps in zones between traditional, motorised and mechanised craft. Existing marine fishing regulations for the respective regions were also evaluated and violations were mapped. Results were presented in each of the villages and then in district wide meetings with community leaders to spur discussions on resource based conflicts and fisheries management. Our findings suggest that the present trajectory of resource over-exploitation, the use of destructive fishing methods combined with the lack of compliance to current regulations will lead to a collapse of the small scale fishing industry and further intensify conflicts within the community. Recommendations made by fishing community leaders are presented and their role in local fisheries management is discussed. This study is the first of its kind for this region and can easily be replicated at regional scales to develop a better understanding of the spatial extent and nature of small scale fisheries, including conflict, for the purpose of fisheries management.

## Introduction

Small scale fisheries employ 90% of the people within the capture fisheries sector either directly or indirectly providing for over 200 million livelihoods [1, 2]. They contribute nearly 60% to the global fish catch, landing close to 22 million tonnes in 2010 alone [3] and play a significant role in poverty alleviation, food security and the provision of livelihoods [4]. Despite this, management of small scale fisheries remains a major challenge. Several studies have established that wherever small scale fisheries are dominant, they are over exploited because of high fishing capacities and are rife with social and economic conflict arising from competition over a common pool resource [5–9].

The small scale fisheries sector is characterised by highly labour intensive operations which reflect comparatively lower capital investments and varying degrees in the utilisation of technology. The term “small scale” includes artisanal and traditional fisheries as well as more modern motorised and mechanised fisheries and exhibits diversity in terms of communities, catch, seasons and fishing techniques employed. Another prominent characteristic of the small scale fishing sector is the numerous formal and informal organisational or institutional arrangements. These often differ across countries and even within the same country [10, 11]. Consequentially, small scale fisheries need to be considered within their specific socio-economic, cultural and ecological context for the purpose of management [12, 13].

Most management discourse follows the assumption that all “artisanal” fishing occurs within 50 nautical miles or over the continental shelf. But what was once “artisanal” has given way to massive fishing capacities and far more intensive extraction, almost comparable to industrial scale fishing [14, 15]. For example, total length of gill nets used in Peru, measured 14 times the length of drift nets used by the Taiwanese high seas fishing fleets while Peruvian long line vessels reportedly set approximately 80 million hooks per year [14]. There is a need to quantify fishing effort and understand where it is employed in order to understand its impacts and to prevent over fishing [16, 17]. The true extent of the impacts of these fisheries is not known as most exist in developing countries particularly in Africa, Latin America and Asia which lack the resources for monitoring and are therefore poorly documented [16]. In comparison, vessel monitoring systems are mandatory in developed nations such as the EU where fishing vessels are required by law to transmit their position, vessel speed and course information in addition to maintaining log books of catch weight, gears and effort employed. Even though, there may be a few constraints related to accessing the data, it is of tremendous value to fisheries management [18–21].

While small scale fishing effort tend to be close to the village/community of origin, fishing capacity and effort cannot be assumed to be distributed evenly. This is particularly when the fisher’s decisions are shaped by the need to share fisheries stocks in heterogeneous coastal ecosystems comprised of a variety of habitats [22–24]. A regional and community analyses of fishing capacities and effort is therefore essential for any cohesive management of local stocks which can be extended to areas lacking sufficient data [25, 26]. Spatial overlaps in fishing territories either by sector or gear, areas of high fishing densities, and potential cross gear effects on the ecosystem are key areas that managers can focus on [27–29]. Following this, maps presenting such information are highly useful in understanding the impacts of fishing by facilitating visualisations of high density fishing zones or violations of existing regulations which in turn are highly instructive to designing management that is more applicable [30–34].

Variations of community based management within adaptive management frameworks have been recommended as the way forward within the small scale fisheries sector [35–37]. Experiences from South East Asia demonstrate reduced conflict and increased food security as direct outcomes of such efforts [8, 38–41]. Other examples include the design, implementation and management of marine protected areas and no take marine reserves. This, along with access rules and the development of local fishing rights has been particularly successful with some small scale fishing communities [42–46]. The prevalence of traditional management systems within the community suggests there is potential for developing an adaptive, scientific, community based management approach that can be locally enforced by fishing communities [26, 47–49].

In India, livelihoods of about four million people depend on marine fisheries of which about one million are active fishers. The mechanised, motorised and artisanal sectors employ about 33, 62 and five percent respectively of these active fisherfolk and account for 75, 23 and two percent respectively of the total production [50]. The spatial expansion of fisheries in India began in the early 1950s when the “blue revolution” brought with it the introduction of trawling and large scale motorisation of traditional craft resulting in increased catches [51]. This rapid expansion of fishing fleets led to over exploitation of stocks within three decades and the phenomenon of “fishing down the food web” was evident across the country. Fishing down the food web refers to when lower trophic level species form the bulk of the catch as the larger higher trophic level species have all been fished out [52].

Indian coastal fisheries have been under tremendous pressure for decades with only a few isolated examples of community based management, primarily in villages where village councils or panchayats existed [53–57]. Conversely, management efforts by the government were mostly in the form of regulations such as the Tamil Nadu Marine Fisheries Regulation Act (1983) and Pondicherry Marine Fisheries Regulation Act (2008) with several amendments and accompanying rules and regulations. All of these are largely ignored [58, 59]. State law was developed mainly to address increasing conflicts between the traditional and trawl fishermen indicating the Government’s unwillingness to get involved until issues reached alarming proportions [52, 60].

Our study was situated in this backdrop of high levels of dependence on a depleting fishery resource, rampant violations of regulations and immense fishing pressure along the coast. We had two key objectives. The first was to develop a spatially explicit database that recorded where fishing occurred in addition to verifying the compliance of Government regulations by the fishing community along the Coromandel Coast of India. The second objective was to engage the fishing community on their thoughts for local fisheries management via a series of co-management meetings in order to develop recommendations for local management.

## Material and methods

### Ethics statement

This research was approved both by the board of trustees and the academic advisory board in the Foundation for Ecological Research, Advocacy and Learning (FERAL). The proposal was also reviewed by an expert committee from the Department of Science and Technology, Science for Equity, Empowerment and Development (SEED) division. We received written consent from fishermen regarding the installation of GPS units on their boats. All other respondents volunteered to be part of the study and had verbally agreed to respond to our queries. Our surveys are anonymous and respondents cannot be identified based on the filled survey forms. We did not require a permit to conduct the study as none of the work was in restricted or protected areas. However, permissions were sought and granted by local community representatives prior to the study. Also, results of the study were shared multiple times with the respondents, both in villages and at collected meetings at the district level. Their feedback is an important part of this study.

### Study area

**Fig 1:**
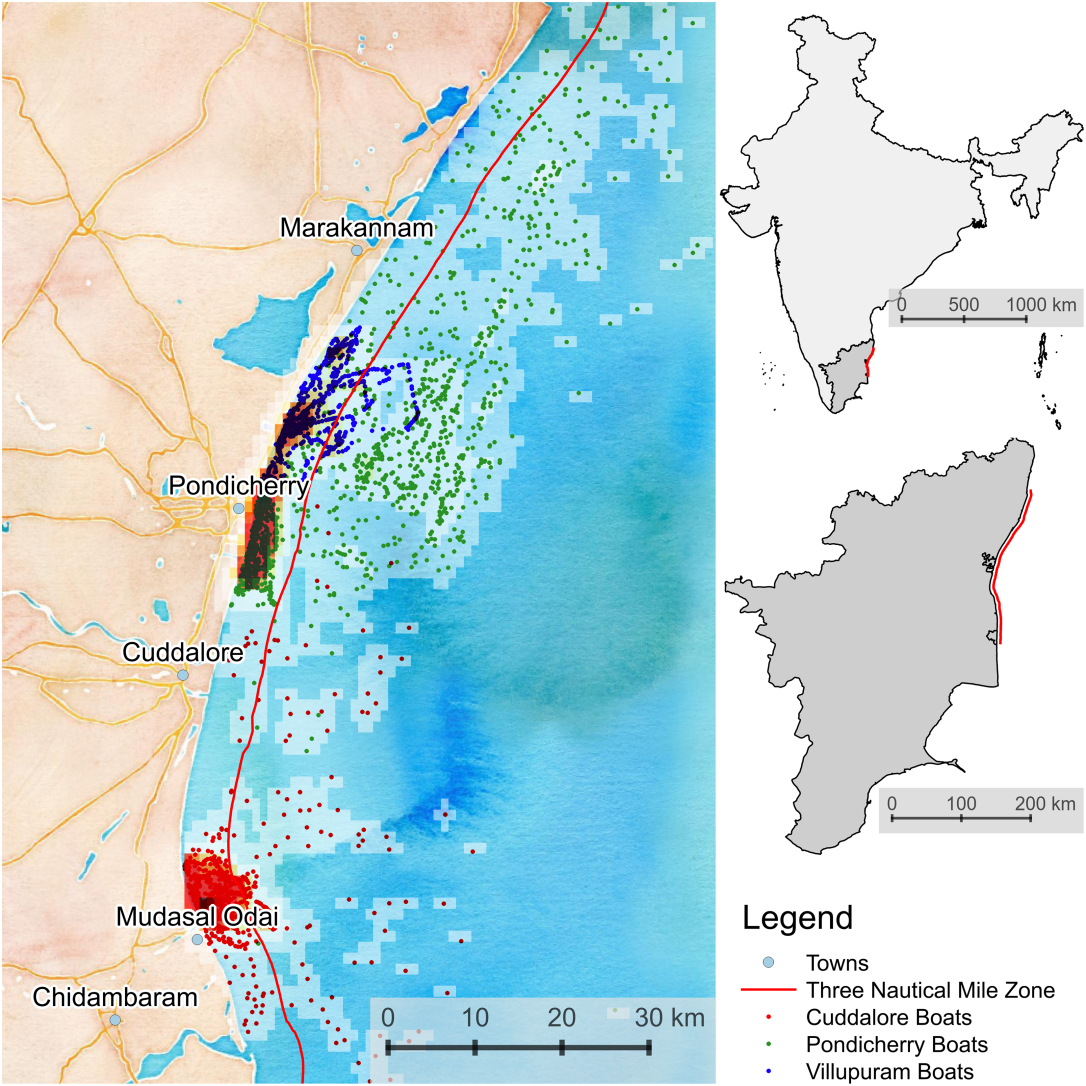
Study area. Map of the study area [61]. Each point represents the location of a fishing event, i.e. a craft operating specific gear. Note that some fishing events engage multiple craft. It is not known why there were fewer observed craft off the Cuddalore coastline.

The coastal waters along 120 km. off the Coromandel Coast were surveyed for fishing activity between June 2012—June 2013 using a combination of coordinates or way-points collected using a global positioning system (GPS) and a structured form for recording observations. This area falls under the administrative divisions of Villupuram and Cuddalore in Tamil Nadu and the Union Territory of Pondicherry (Fig 1). Table 1 presents details of the type of craft, gear and associated mesh sizes and crew sizes commonly seen in this region.

**Table 1:** Summary of craft and gear used along the study area. Traditional craft comprise Kattumarams or wooden rafts which are not powered by engines and are rowed out to sea. Small and big fibreglass reinforced plastic (FRP) boats are motorised craft powered by outboard engines of varying horsepower (HP). The mechanised craft comprise trawlers and vallams with more powerful inboard engines (S1 Fig). There is an overlap in the type of gear used by each category of fishing craft. Gill nets in particular varied in nomenclature according to the village resulting in a broad classification which comprises mono-filament and multi-filament nets and a small proportion of trammel nets. Similarly, trawl nets used by the motorised craft were considered “modified” trawls and variations of the ring seine were also observed. Large crew sizes particularly for lift nets, gill nets and trawl nets by the motorised craft indicate more than one boat per operation. Ring seines also involved collaborations among and between motorised fibreglass boats along with the mechanised larger craft called vallams.

Category	Type of craft	Overall Length (m)	Motor Power (HP)	Type of gear & fishery	Mesh/hook size range (mm)	Crew size range
Traditional	Kattumarams or wooden rafts	^~^3.5	NA	Gill nets	14–180	1–8
Traditional	Kattumarams	^~^3.5	NA	Hook & Line	7–16	1–2
Motorised	Small FRPs	^~^7	^~^10	Gill nets	5–300	1–28
Motorised	Small FRPs	^~^7	^~^10	Hook & Line	7–16	1–6
Motorised	Small FRPs	^~^7	^~^10	Lift nets	12–40	4–45
Motorised	Small FRPs	^~^7	^~^10	Ring seines	14–76	10–45
Motorised	Small FRPs	^~^7	^~^10	Scoop nets	14–34	3–6
Motorised	Small FRPs	^~^7	^~^10	Trawl nets	16–40	1–5
Motorised	Big FRPs	^~^7-9	^~^16	Gill nets	18–128	2–5
Motorised	Big FRPs	^~^7-9	^~^16	Hook & Line	14–16	^~^ 4
Motorised	Big FRPs	^~^7-9	^~^16	Ring seines	18–44	20–50
Motorised	Big FRPs	^~^7-9	^~^16	Trawl nets	20	3–14
Mechanised	Trawlers	14	^~^72-472	Hook & Line	^~^ 6	^~^ 6
Mechanised	Trawlers	14	^~^72-472	Trawl nets	20–40	3–14
Mechanised	Vallams—large carrier craft	22	^~^472	Ring seines	18–110	25–65

### Data collection

Fishing activity in approximately 7,945 sq. km. of the study area was surveyed using nine Garmin GPSMAP 585 chart plotters (Fig 1). These units comprise a high-sensitivity GPS module with a horizontal accuracy of about 4 meters. The GPS is coupled with a sonar unit comprising a transducer operating at a frequency of 50/200 kHz. The sonar can operate up to depths of 450 m (1500 ft) and records depth contours, fish targets and structures below the surface including substrate type, broadly characterised as sandy, clay, muddy or rocky mixtures. Similar equipment is used both in commercial and sport fishing and is designed to withstand extreme conditions. Thus each way-point recorded by the GPS comprised of the x, y and z coordinates (depth) and the type of substrate.

Fishermen who owned mechanised craft i.e., trawlers and vallams volunteered to provide location data using these units and eight of them were fitted onto six trawlers and two vallams. Tracks of the craft movement for the entire duration of the fishing trip were captured automatically by the GPS. Fishermen volunteers manually recorded way-points whenever nets were deployed. These fishing trips ranged from one to five days and data regarding the gear and catch were collected from these craft either upon their return to the harbour or every fortnight. A total of 1,257 observations were made by these volunteers which included multi-day fishing trips.

One GPS/sonar unit was used by field team members to record data on craft actively fishing in the region. This was done in collaboration with fishermen on a small fibreglass boat for three cycles of data collection. Each cycle comprised of five consecutive days in each region i.e., two districts and the state of Pondicherry, completing 15 days of sampling per cycle. Boat surveys were spread seasonally between pre-monsoon (August–October), post monsoon (December–March) and summer (April–July). Surveys coincided with the time when fishermen routinely began their fishing trips each day in an attempt to record as many active fishing boats from that respective region. We made a total of 2,170 observations wherein the survey boat was taken within five metres of the fishing craft to mark a way-point and record details on type of craft (Table 1), type of gear, mesh size, crew size and the village of origin by direct observation and interaction with respective fishermen.

Thus, each data point contains records of a single fishing operation by a craft. A total of 3,427 fishing operations (volunteers + field team) were recorded which included traditional, motorised and mechanised craft (Table 2). Some gear, such as ring seines had large crews and involved multiple craft in a single fishing operation. These were listed as a single observation (Table 3) therefore the total number of fishing boats observed during the study was 5,003 which is more than the number of observations (fishing operations) recorded. The coordinates of the largest craft were used as the observation point for multi-craft operations.

**Table 2:** Total number of fishing operations recorded over the study period listed by type of gear. A large portion of the data collected for ring seines and trawls came from the units fitted onto the fishing vessels (6 trawlers and 2 vallams). The remaining gear operations (lift nets, hook and lines, gill nets, scoop nets) including some ring seines and trawlers, were observed and recorded during the three cycles of data collection by the field team.

Type of gear	No. of observations
Lift nets	4
Hook & Line	69
Gill nets	1762
Ring seines	213
Scoop nets	134
Trawl nets	1245
Total	3427

**Table 3:** Total numbers of gear and craft employing each gear type along with associated collaborations observed at sea. This is particularly evident between ring seines and a few gill nets. Ring seines, trawls, lift nets and a few gill nets also employed more than one boat which explains the higher craft to gear ratio. This table represents the type of collaborations observed at sea and is not an exhaustive list of all the collaborations that may exist for this region.

Type of gear	No. of gear	Primary craft type	No. of boats	Collaborating craft type	No. of boats
Gill nets	728	Kattumarams	728	--	--
	1001	Small Fibreglass boats	1035	Kattumarams	2
	33	Big Fibreglass boats	33	--	--
Lift nets	4	Small Fibreglass boats	17	--	--
Lines	25	Kattumarams	25	--	--
	4	Small Fibreglass boats	4	--	--
	40	Trawlers	40	--	--
Ring seines	29	Small Fibreglass boats	146	--	--
	184	Vallams	184	Small Fibreglass boats	1381
Scoop nets	134	Small Fibreglass boats	134	--	--
Trawl nets	62	Small Fibreglass boats	62	--	--
	3	Big Fibreglass boats	3	--	--
	1180	Trawlers	1209	--	--

### Data entry and analysis

We used PostgreSQL [62] and PostGIS [63], a spatial relational database to store and organise the data. The GIS packages GRASS GIS [64], and Quantum GIS [65] were used for geo-processing as well as preparing maps. The R package for statistical computing [66] was used to visualise and statistically analyse spatial and non-spatial relationships between observations. Non parametric techniques were used as the data were not normal. The R libraries used for analysis and visualisation included packages spatstat [67], raster [68], spdep [69], dunn.test [70], doBY [71], gridExtra [72], ggplot2 [73] and plotly [74] (R script with documentation and datasets are provided in S1 File).

### Mapping fishing zones and densities

Way-points and attributes (S1 Table and S2 File) collected during the fishing surveys were merged to create a point GIS layer where each point corresponds to the coordinates of an observed fishing operation and its attributes correspond to observations recorded such as type of gear and craft, gear mesh size, total number of boats, crew size, bottom depth and substrate. A recent very high resolution satellite image was used to digitise the shoreline and the shortest distance from the shoreline to each observation was added to the attribute table using the “v.distance” module in GRASS. Two dimensional kernel density estimates with contours using the “stat_density2d” function of the ggplot2 package were then used to visualise the intensity of fishing effort across different combinations of craft and gear.

### Spatial patterns in fishing

We used the local Geary’s test to determine spatial auto-correlation between numeric variables to examine if gear with similar mesh sizes or boats with similarly powered engines were likely to occur together. The Getis-Ord global G statistic was then used to compute clustering between high values and low values of gear mesh size data and to determine whether smaller or larger mesh sizes were spatially clustered. Join counts were used to compare degrees of spatial correlation between and within categorical groups, such as type of craft and gear. This allowed us to identify whether specific types of craft and gear were using spatially distinct zones for fishing. A join count significantly lower than the expected count implies positive spatial auto-correlation or clustering while negative spatial auto-correlation (dispersion) is when joins are significantly higher than expected. Spatial randomness occurs when the joins are similar to the expected value.

The Kruskal-Wallis (KW) test was used to determine if there were significant differences between craft and gear with respect to fishing densities and fishing zones. The KW tests were followed by *post hoc* multiple comparison tests that use pairwise comparisons and employ the False Discovery Rate (FDR) method to reduce the incident of false positives due to repetitive testing. Comparisons of medians were visualised via simple box and whisker plots.

### Mapping and quantifying violations

We also compared existing marine fishing regulations between the state of Tamil Nadu and Pondicherry in terms of the number of violations observed. Regulations were related to distance of fishing from the shore, gear mesh sizes, size of craft, engine horsepower and whether craft were used for multi-day fishing. The shortest distance to shore was used to determine whether the location of observed craft complied with regulations on fishing zones. All other attributes were directly compared against stipulations as per the respective regulations and violations were recorded as percentages of the total number of boats observed.

## Results

### Fishing distances, bottom depths and mesh sizes

A summary of bottom depths, fishing distances and mesh sizes used by each fishery as characterised by gear and craft is presented in Tables 4 and 5.

**Table 4:** Summaries of fishing zones and mesh/hook sizes employed across the fisheries of the study area. Gill and trawl nets were most commonly encountered. Also trawls and ring seines were operated at similar median depths and distances. Line fisheries were furthest from the shoreline while the scoop nets were the closest.

Fishery	No. of observations	Median Depth (m)	Min	Max	Std. Dev
Gill net	1762	12.25	3.47	33.47	5.19
Lift	4	14.08	11.7	17.56	2.49
Line	69	24.51	4.39	107.72	21.62
Ring seine	213	24.69	2.74	110.09	21.5
Scoop net	134	8.05	2.56	13.35	2.38
Trawl	1245	24.51	3.47	103.69	15.99
Fishery	No. of observations	Median Distance to shore (km)	Min	Max	Std. Dev
Gillnet	1762	1.82	0.03	13.4	2.43
Lift	4	4.11	1.98	5.62	1.61
Line	69	13.26	0.11	36.09	11.21
Ring seine	213	10.3	0.22	82.32	8.27
Scoop net	134	0.8	0.1	16.68	1.53
Trawl	1245	10.88	0.14	81.94	8.45
Fishery	No. of observations	Median mesh size (mm)	Min	Max	Std. Dev
Gillnet	1759	46	6.6	120	16.65
Lift	4	12	12	12	0
Line	69	6	6	15	3.95
Ring seine	213	28	16	110	37.74
Scoop net	134	12	12	12	0
Trawl	1245	20	20	60	1.39

**Table 5:** Summaries of fishing zones and mesh/hook sizes employed by fishing craft of the study area. Most observations were of small FRP craft and trawlers. Trawlers operated at the same minimum depths as the traditional kattumaram. Median distances of both trawlers and vallams were very similar. Trawlers operated the smaller median mesh sizes followed by vallams.

Type of craft	No. of observations	Median depth (m)	Min	Max	Std. Dev
Small FRP	1234	13.72	2.56	33.47	5.67
Big FRP	36	16.09	7.68	26.88	4.34
Kattumaram	753	9.69	3.47	23.59	2.8
Trawler	1220	25.24	3.47	107.72	16.17
Vallam	184	26.7	8.05	110.09	21.6
Type of craft	No. of observations	Median distance to shore (km)	Min	Max	Std. Dev
Small FRP	1234	2.59	0.1	16.68	2.64
Big FRP	36	3.98	0.52	7.87	1.68
Kattumaram	753	0.88	0.03	10.8	1.06
Trawler	1220	11.36	0.14	81.94	8.51
Vallam	184	11.45	0.49	82.32	8.09
Type of craft	No. of observations	Median mesh size (mm)	Min	Max	Std. Dev
Small FRP	1232	52	7	120	20.71
Big FRP	36	75	18	112	25.86
Kattumaram	752	44	6.6	75	12.74
Trawler	1220	20	6	20	2.49
Vallam	184	34	18	110	38.84

**Fig 2:**
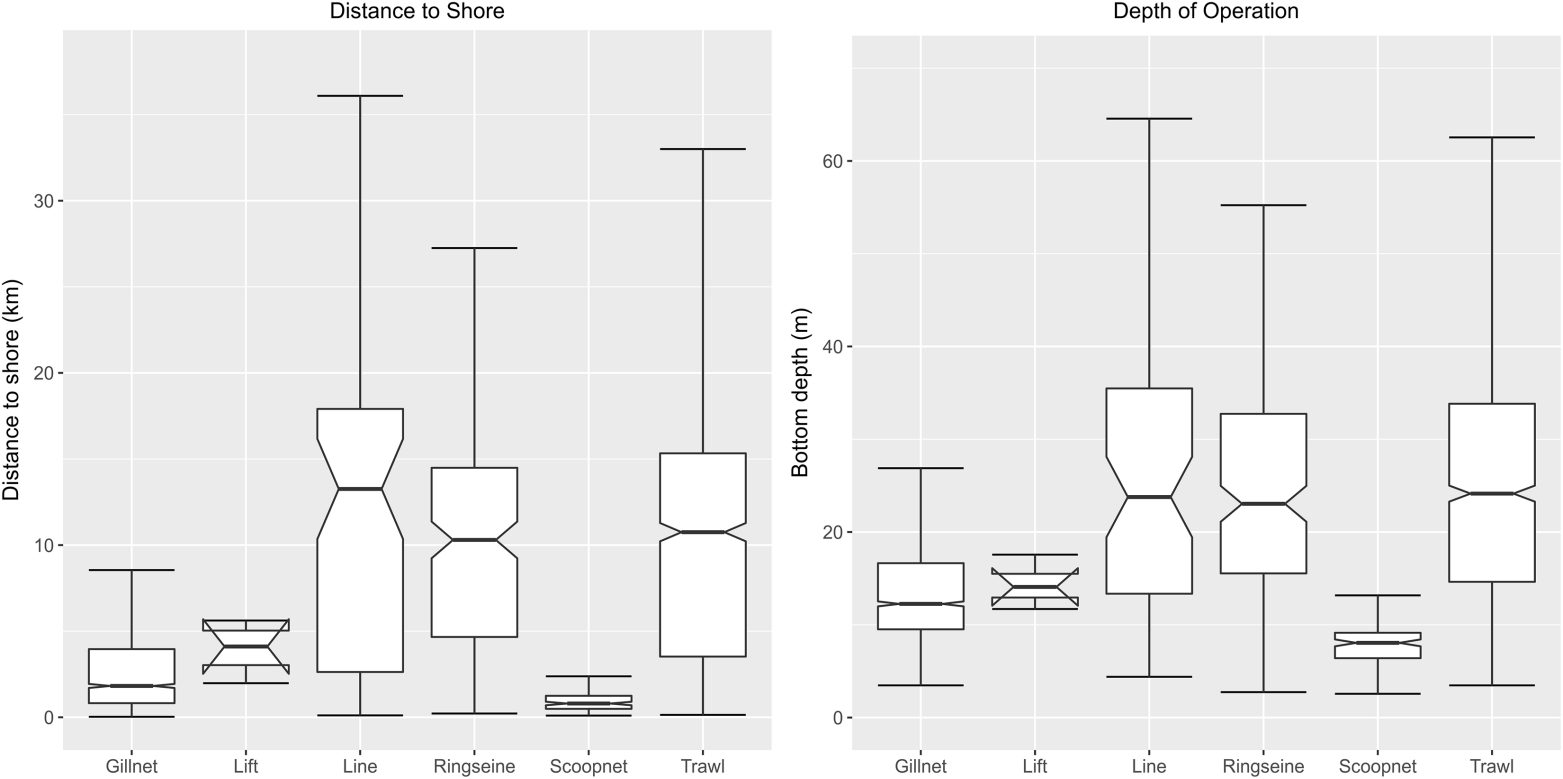
Fishing gear versus distance from the shore and bottom depths. Trawl, line and ring seines utilise a similar and fairly wide range of distances from shore and fishing depths. Scoop nets appear to be occupying a very specific niche close to the shore and in shallow depths while gill and lift nets are used further offshore within a fairly narrow range of depths and distances. Outliers have been excluded to visualise the spread of the data.

Fishing distances and bottom depths were significantly different between each fishery (KW chi-squared = 1111.5, df = 5, p-value <2.2e-16). Scoop nets and lift nets were recorded fishing within 5–10 km of the shore at bottom depths within 20 m. Gill nets fished within 10 km of the shore at bottom depths below 30 m. Lines, ring seines and trawls operated in wider ranges of depth and distance from the shore i.e., within 25 m and 10–15 km respectively (See Fig 2). *Post hoc* tests after KW revealed significant differences between scoop nets and all other fishing gear (FDR adjusted p.value <0.05). Gill nets were also significantly different from all gears except lift nets (FDR adjusted p-value <0.05). Trawls and ring seines were significantly different from gill nets and scoop nets. No significant differences were found between trawls and ring seines (Table 6).

**Table 6:** Spatial auto-correlation tests using local Geary’s C statistic. Values closer to 0 indicate strong positive auto-correlation while values closer to 1 indicate random distribution with no auto-correlation. Distances to the shore-line and to a lesser degree, depths, have the strongest auto-correlation, clearly demonstrating a preference for near-shore fishing grounds.

Variable	Geary C Statistic	Expectation	Variance	Adjusted p.value
Distance to shore (km)	0	1	0.00083	<2.2e-16
Bottom depth (m)	0.18	1	0.00067	<2.2e-16
Length of craft (ft)	0.19	1	0.00049	<2.2e-16
Engine Horsepower	0.23	1	0.00046	<2.2e-16
No. of crew	0.34	1	0.00082	<2.2e-16
Mesh sizes (mm)	0.45	1	0.00054	<2.2e-16

**Fig 3:**
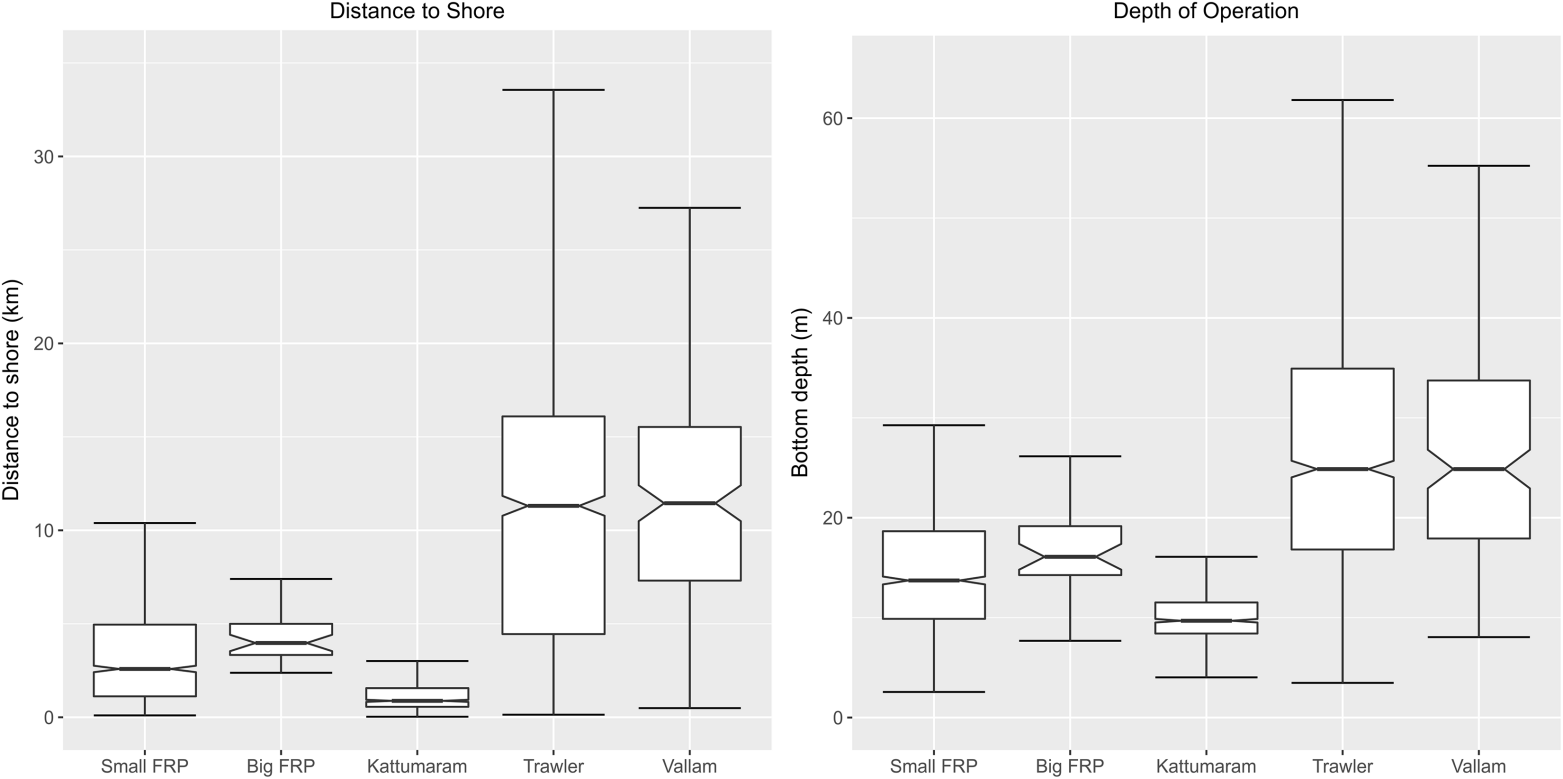
Craft type versus distance from shore and bottom depths. The mechanised trawlers and vallams utilise a similar and fairly wide range of distances from shore and fishing depths. Traditional kattumarams occupy a very specific niche close to the shore and in shallow depths while the motorised FRP boats are used further offshore in similar depths and distances, also in a narrow range. Outliers have been excluded to visualise the spread of the data.

Fishing craft also displayed significant differences in terms of fishing distances and boat type (KW chi-squared = 1713.8, df = 4, p-value <2.2e-16; Fig 3). Mechanised craft (trawlers and vallams) fished at greater distances and bottom depths while motorised and traditional craft fished comparatively closer to shore (<5 km) and at shallower depths (10–20 m). *Post hoc* tests after KW showed significant differences in fishing distances and bottom depths between each craft type. Only trawlers and vallams were not significantly different in terms of fishing distances (FDR adjusted p.value <0.05).

**Fig 4:**
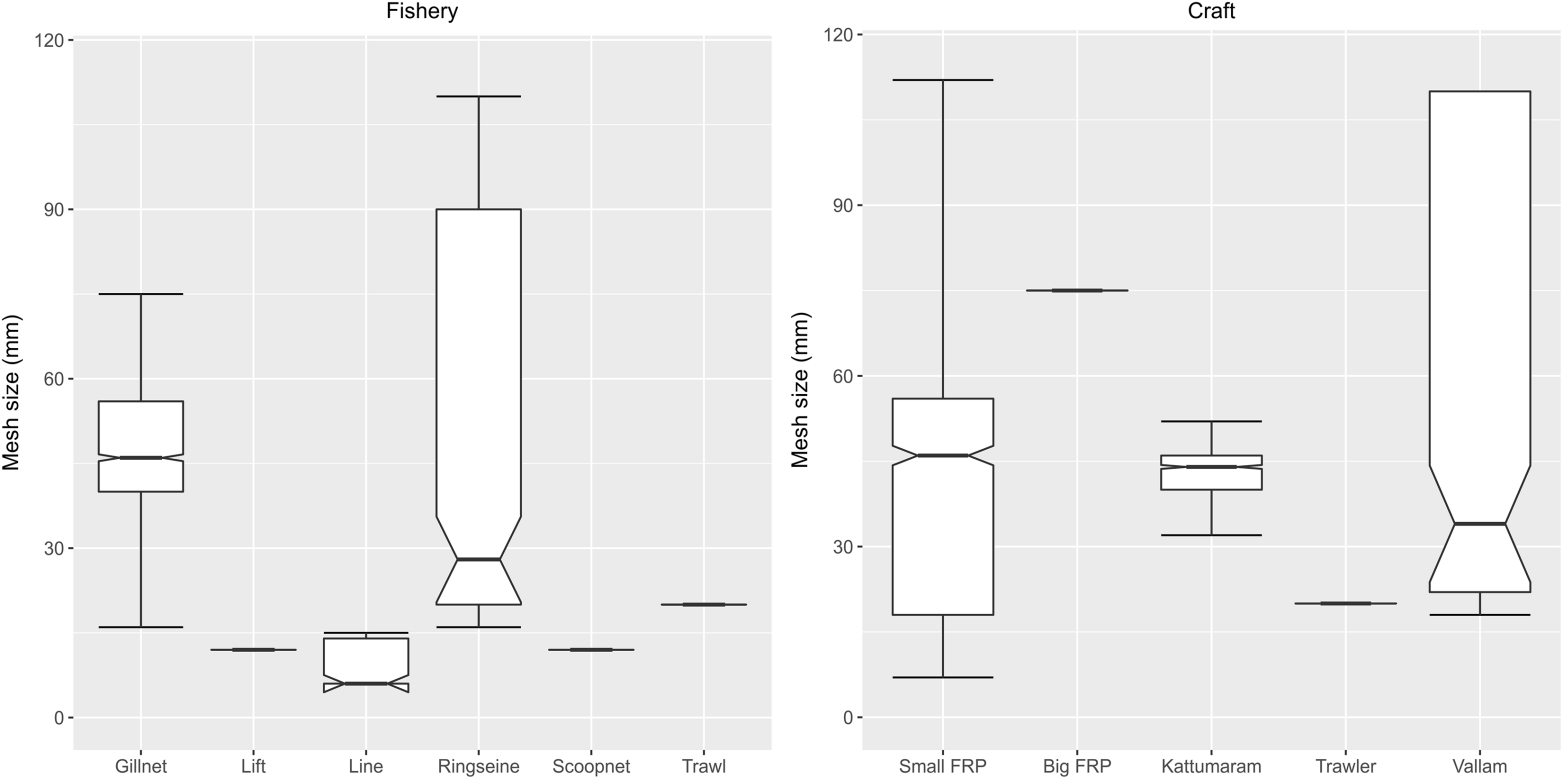
Mesh size versus gear and craft type. All the fisheries except gill nets and ring seines appear to operate a distinct and fairly narrow range of mesh sizes. The mesh size of ring seines overlaps with gill nets however, ring seines have a much large range of mesh sizes. Kattumarams, small FRPs and vallams operate similar mesh sizes, however the range of mesh sizes operated is substantially larger in small FRPs and even more so with vallams. Trawlers operate nets with the smallest mesh sizes while big FRPs operate the largest, both within a narrow range. Note that values for line fisheries is size of hooks. Outliers were excluded to visualise the spread of the data.

Mesh sizes were significantly different between each fishery (KW chi-squared = 1756.1, df = 5, p-value <2.2e-16) and craft (KW chi-squared = 953.14, df = 4, p-value <2.2e-16). Gill nets and ring seines displayed a wider range of mesh sizes, the former employed by small motorised boats and the latter by vallams. Lines had a median hook size = 6 and also displayed a wider range in comparison to lift nets, scoop nets and trawls which were limited in range and had lower mesh sizes (Fig 4). *Post hoc* tests after KW computed significant differences between gill nets and all other gear (FDR adjusted p.value <0.05). Ring seines were also significantly different from all other gear (FDR adjusted p.value <0.05). No significant differences were evident between mesh sizes employed between kattumarams and vallams. All other fishing craft employed significantly different mesh sizes (FDR adjusted p.value <0.05).

### Fishing densities and locations

**Fig 5:**
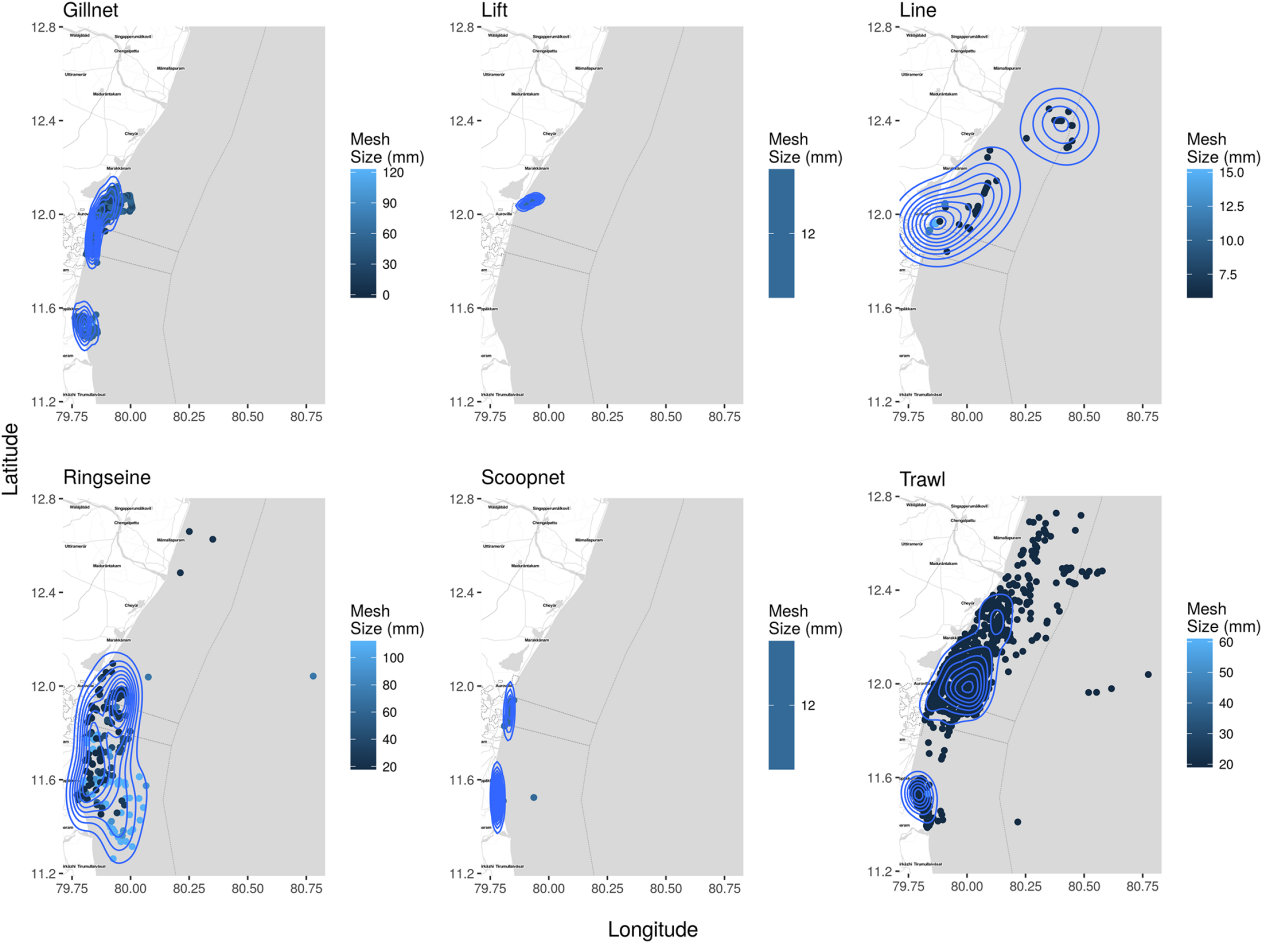
Kernel density estimates and location of type of gear and mesh sizes of gear they operate. Each dot represents an observed craft and the darker the dots, the smaller the mesh/hook size. The contours visualise the density of fisheries and can be used as a proxy for fishing effort per type of fishery. The region off Cuddalore was only used by ring seine fisheries. Scoop and gill nets were concentrated very close to the shore, while trawl nets had the largest spread followed by ring seines. Scoop nets were only observed at Villupuram and line fishing, which had the largest spread, was observed north of Pondicherry and in deeper waters. An interactive plot is provided in S3 File.

**Fig 6:**
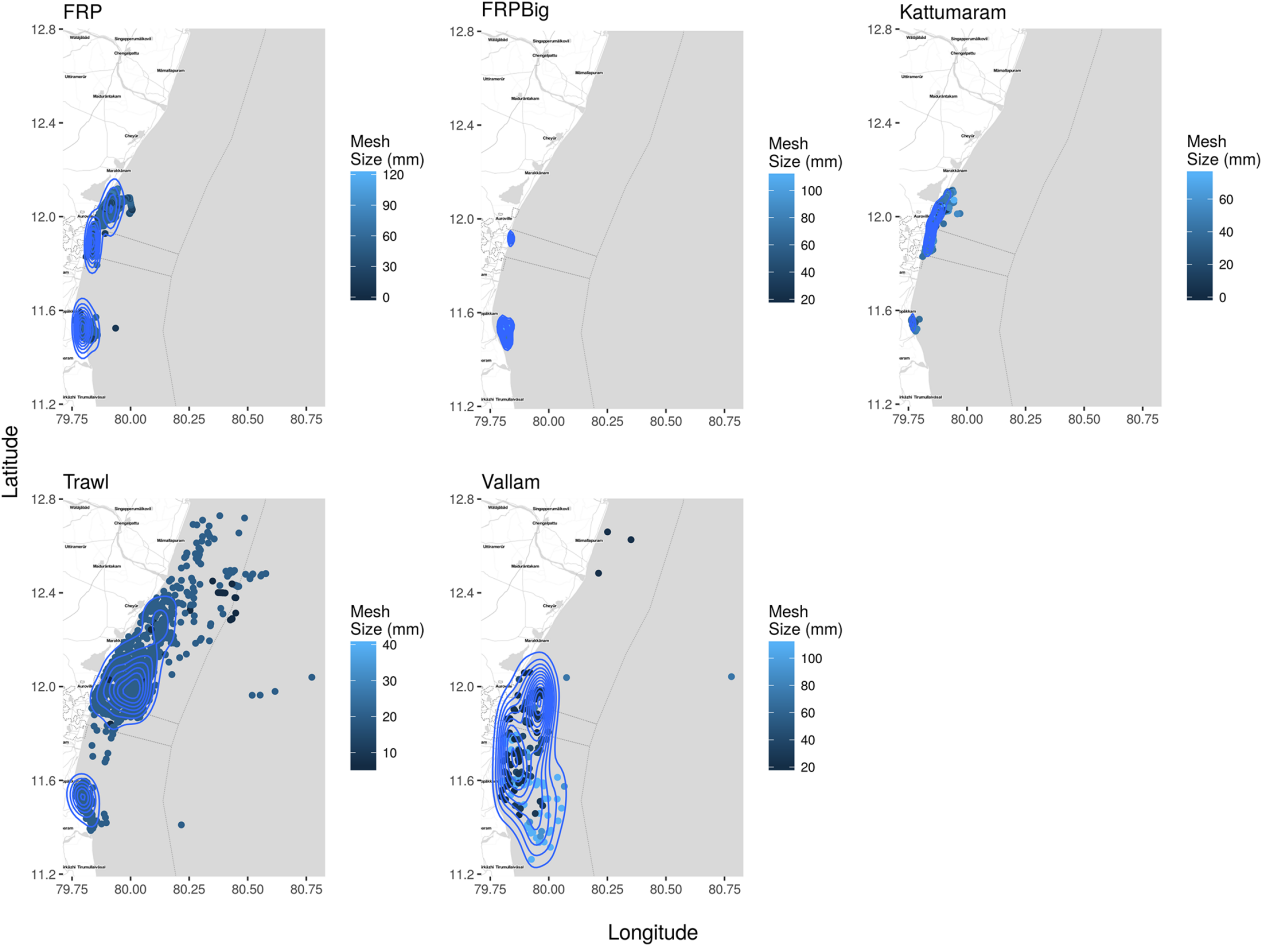
Kernel density estimates and location of craft type and mesh sizes of gear they operate. Each dot represents an observed craft and the darker the dots, the smaller the mesh/hook size. The contours visualise the density of craft and can be used as a proxy for fishing effort per craft type. Only vallams were seen to operate in the region off Cuddalore. Trawls covered the largest area followed by vallams, however most of these mechanised craft operated well within the three nautical mile zone. The artisanal and motorised craft operated very close to the coastline. An interactive plot is provided in S4 File.

**Fig 7:**
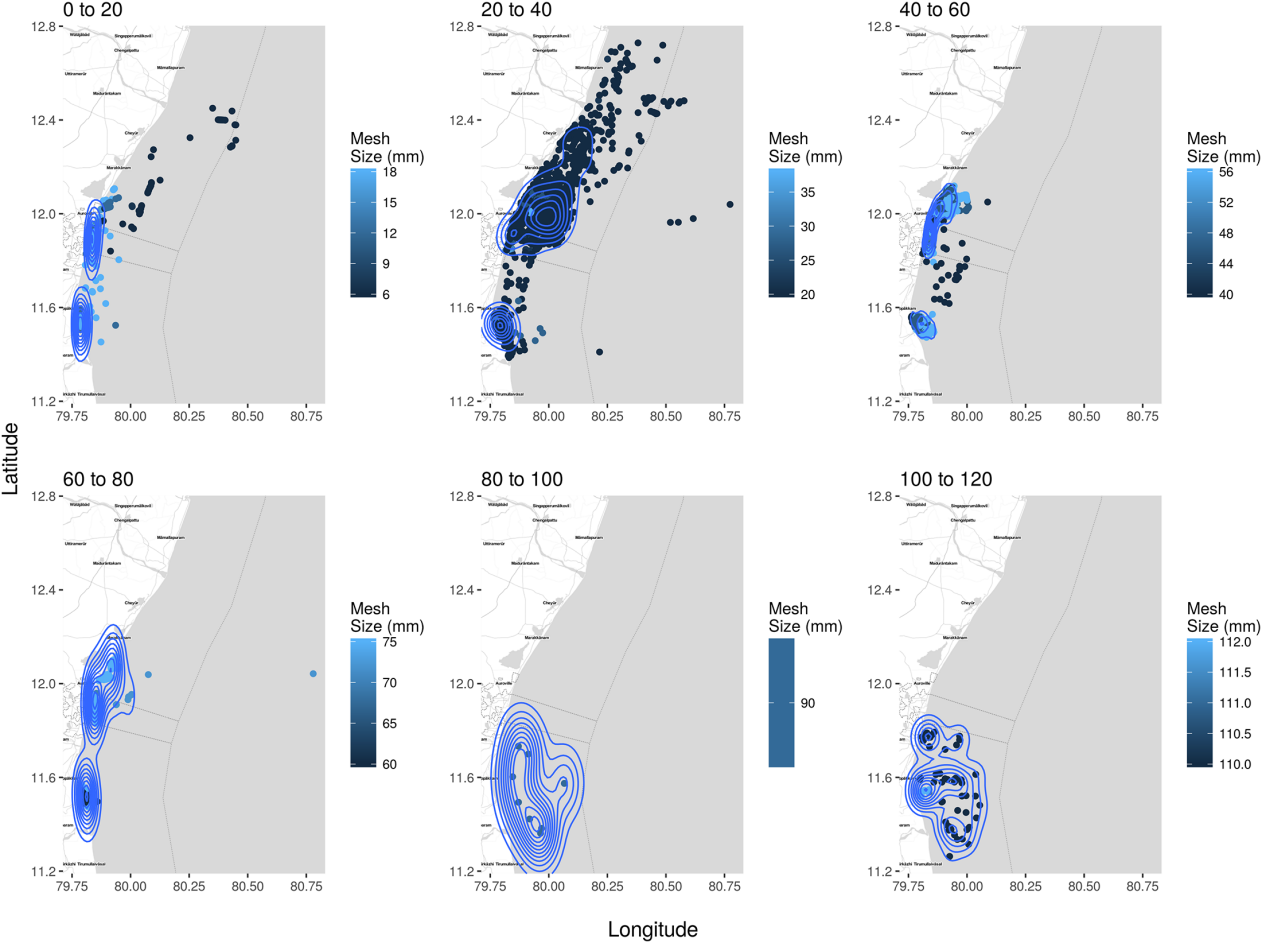
Kernel density estimates and location of all craft and mesh size classes of gear they operate. To better visualise the wide range of mesh sizes used along the study area, we grouped the data into six size classes. Each dot represents an observed craft and the darker the dots, the smaller the mesh/hook size. The contours visualise the density of each class of mesh sizes operated, across all fisheries and can be used as a proxy for fishing effort per mesh size class. The largest mesh size classes are operated near Cuddalore and are also used in deeper waters. Most fisheries are operating mesh sizes between 20 and 40mm and are located off Cuddalore, Pondicherry and to the north of Pondicherry. An interactive plot is provided in S5 File.

Maps of cumulative fishing densities present the concentration and distribution of fishing effort for each fishery as defined by the type of gear (Fig 5) and associated craft type (Fig 6). Gill nets had the highest density closest to the shore, while lines, lift nets and scoop nets were operated in comparatively lower densities and over smaller areas. Distinct zones of high concentrations/densities of smaller and larger mesh sizes are clearly evident (Fig 7).

**Fig 8:**
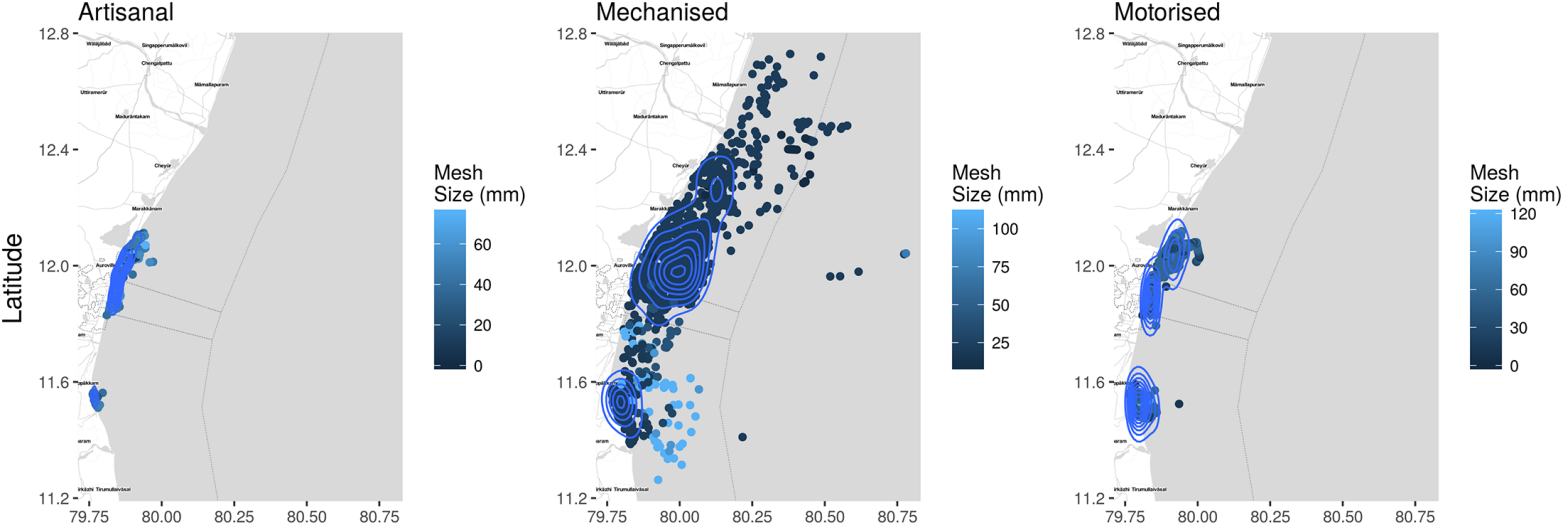
Kernel density estimates and location of different craft classes and mesh sizes of gear they operate. This plot presents the overall distribution of fishing effort along the study area. Each dot represents an observed craft and the darker the dots, the smaller the mesh/hook size. The contours visualise the densities of the different categories of craft operated and can be used as a proxy for fishing effort by class of craft. The proximity of the artisanal and motorised craft to the coast is expected. However mechanised craft also operate in this region, which is a violation of the zonal restrictions and the major cause of conflict between mechanised craft who operate from jetties and harbours and motorised and artisanal craft which operate from coastal villages. An interactive plot is provided in S6 File.

Trawls and ring seines exhibited pockets of high fishing density and were spread over a larger area. Similarly, traditional and motorised craft were highly concentrated near the coast along with trawlers and vallams although mechanised craft were distributed over a larger area (see Fig 8). Large overlaps were observed in fishing zones between each sector and between each gear and craft type.

### Spatial patterns in fishing

The local Geary’s test showed significantly strong spatial auto-correlation for fishing distances from the shoreline, bottom depths, boat lengths and associated engine power, crew sizes and finally mesh sizes (FDR adjusted p.value <0.05, Table 6). The Getis-Ord global G statistic indicated significant clustering between larger (high—high correlation) and smaller (low—low correlation) mesh sizes respectively (G statistic = 4.92e-04, Std Dev. = 17.6, p-value <0.05).

Large overlaps in fishing zones between the three sectors in terms of fishing craft and gear were further confirmed by join count tests (Table 7). Boats from within the same category appeared to be dispersed i.e., no clustering was seen within each respective boat type. However, significant clustering between mechanised craft (trawlers and vallams), motorised (big and small FRP boats) and traditional craft (kattumarams) was evident. Only the bigger motorised boats and vallams appeared to be random and were not spatially auto-correlated.

**Table 7:** Join count test between nominal variables and fishery to test for spatial auto-correlation. Join counts lower than the expected values indicate clustering while join counts higher than expected values indicate dispersion. Join counts that were close to expected values were considered to be random.

Join count between nominal or categorical variable and craft type						
Craft type	Join count	Expected	Variance	z.value	Adjusted p. value	Decision
Kattumaram:Small FRP	179.92	270.66	124.03	-8.15	2.96e-16	Clustered
Kattumaram:Big FRP	1.50	7.91	4.76	-2.94	1.89e-03	Clustered
Trawler:Small FRP	122.08	439.10	186.21	-23.23	2.90e-119	Clustered
Trawler:Big FRP	5.42	12.83	6.58	-2.89	2.05e-03	Clustered
Trawler:Kattumaram	33.54	268.02	123.21	-21.12	5.42e-99	Clustered
Vallam:Small FRP	10.92	66.22	33.10	-9.61	6.21e-22	Clustered
Vallam:Kattumaram	6.25	40.42	23.60	-7.03	1.46e-12	Clustered
Vallam:Trawler	34.42	65.58	32.91	-5.43	3.71e-08	Clustered
Small FRP:Small FRP	448.33	221.53	77.41	25.78	2.54e-146	Dispersed
Big FRP:Big FRP	4.17	0.18	0.14	10.81	3.18e-27	Dispersed
Kattumaram:Kattumaram	267.33	82.49	40.30	29.12	4.57e-186	Dispersed
Trawler:Trawler	509.92	217.23	76.61	33.44	1.47e-244	Dispersed
Vallam:Vallam	64.42	4.92	3.36	32.48	5.34e-231	Dispersed
Big FRP:Small FRP	23.04	12.96	6.62	3.92	5.43e-05	Dispersed
Vallam:Big FRP	0.75	1.94	1.37	-1.01	1.56e-01	Random
Total number of Joins	417.83	1185.64	255.93	-47.99	0	Clustered
Join count between nominal or categorical variable and fishery						
Fishery	Join count	Expected	Variance	z.value	p. value	Decision
Line:Gill net	10.92	35.44	14.53	-6.43	1.53e-10	Clustered
Ring seine:Gill net	30.67	109.39	44.51	-11.80	5.36e-32	Clustered
Scoop net:Gill net	47.04	69.33	28.33	-4.19	2.58e-05	Clustered
Trawl:Gill net	172.29	639.41	245.85	-29.79	1.37e-194	Clustered
Trawl:Ring seine	40.58	77.47	38.38	-5.95	2.61e-09	Clustered
Trawl:Scoop net	17.42	49.10	24.60	-6.39	1.84e-10	Clustered
Gill net:Gill net	750.42	451.18	100.54	29.84	3.99e-195	Dispersed
Line:Line	18.58	0.69	0.50	25.39	6.60e-142	Dispersed
Ring seine:Ring seine	64.42	6.60	4.43	27.47	8.24e-166	Dispersed
Scoop net:Scoop net	32.75	2.64	1.85	22.13	2.50e-108	Dispersed
Trawl:Trawl	491.17	226.23	78.28	29.95	2.80e-196	Dispersed
Lift net:Gill net	4.00	2.05	0.85	2.12	0.03	Dispersed
Lift net:Lift net	0.00	0.00	0.00	-0.05	0.48	Random
Line:Lift net	0.00	0.08	0.06	-0.33	0.41	Random
Ring seine:Lift net	0.00	0.25	0.18	-0.59	0.34	Random
Ring seine:Line	2.00	4.29	3.00	-1.32	0.12	Random
Scoop net:Lift net	0.00	0.16	0.11	-0.47	0.37	Random
Scoop net:Line	0.42	2.72	1.94	-1.65	0.07	Random
Scoop net:Ring seine	4.58	8.40	5.77	-1.59	0.08	Random
Trawl:Lift net	0.00	1.45	0.74	-1.69	0.07	Random
Trawl:Line	24.75	25.10	12.69	-0.10	0.48	Random
Total number of Joins	354.67	1024.66	258.30	-41.69	0.00	Clustered

All fishing gears except for lift nets were highly spatially auto-correlated with gill nets (FDR adjusted p-values <0.05). Significant clustering was seen between trawlers, ring seines and scoop nets respectively (FDR adjusted p-values <0.05). Gear of the same type appeared to be highly spatially dispersed while dissimilar gear appeared to be highly spatially correlated although some gear were not spatially auto-correlated and were random in distribution (Table 7).

### Violations of the marine fisheries regulation acts

**Fig 9:**
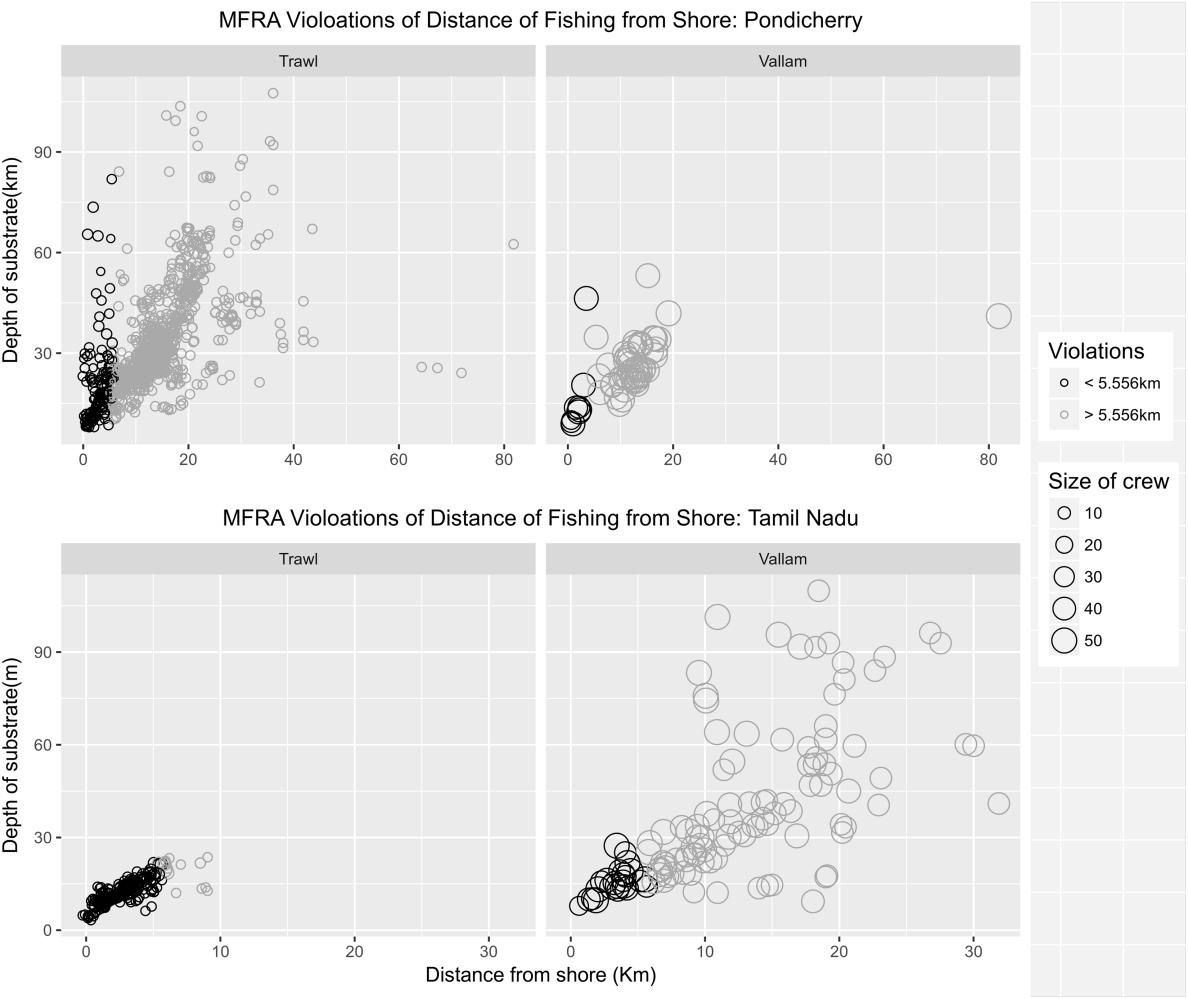
Violations of the three nautical mile (5.556 km) restriction by mechanised craft (trawlers & vallams) from Pondicherry and Tamil Nadu. The black circles indicate violations of the rule. Higher number of violations were evident among trawlers particularly in Tamil Nadu (93%). Pondicherry had relatively fewer violations (15%) compared to Tamil Nadu (68%).

**Fig 10:**
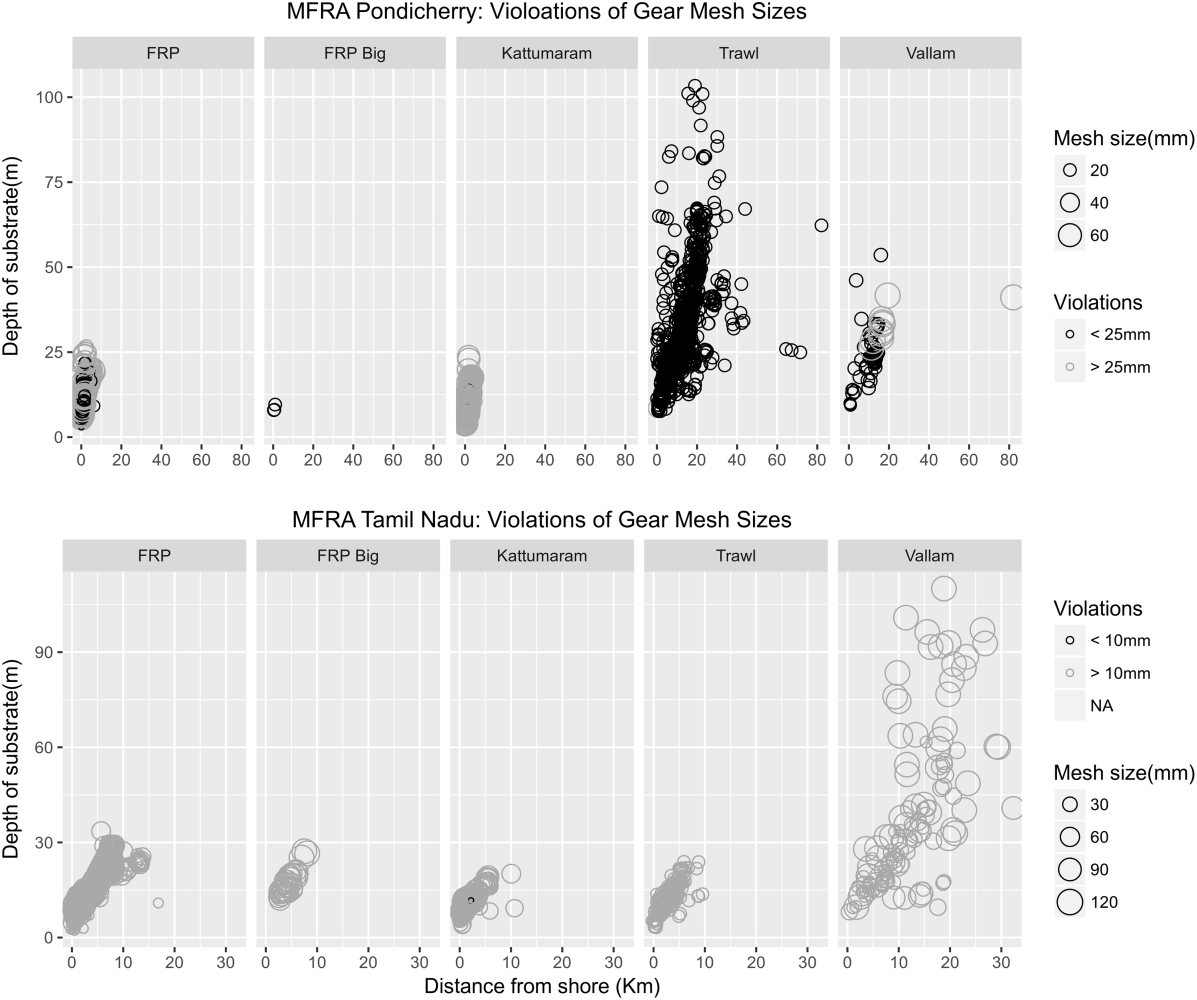
Violations of the mesh size restrictions by all craft from Pondicherry and Tamil Nadu as per MFRA rules of their respective states. The black circles indicate violations of the rule. Higher number of violations were evident in Pondicherry (64%). Tamil Nadu had fewer violations primarily because the minimum mesh size for all gears except trawlers is set at just 10 mm (0.2%).

Large scale violations of the Marine Fisheries Rgulation Act (MFRA) were observed particularly with reference to the three nautical mile fishing limit, legal mesh sizes and the use of illegal fishing gear (Table 8). The fishing regulation acts of both Tamil Nadu and Pondicherry clearly specify engine power and boat length typical of mechanised craft and deep sea craft. Consequently, 76% and 35% of the mechanised craft recorded fishing in Pondicherry and Tamil Nadu respectively would be classified as deep sea vessels and would be in violation of fishing distances specified for deep sea vessels. While length restrictions were mostly followed, <5% engine horsepower violations were common among the bigger motorised boats, trawlers and vallams. About 66% of the trawlers in Pondicherry fished for multiple days beyond the three nautical rule without returning to the harbour and were therefore in violation of the rule. Furthermore, a higher number of violations of the three nautical mile rule by mechanised craft were recorded in Tamil Nadu (68%) than in Pondicherry (15%); 93% of the trawlers recorded in Tamil Nadu fished within three nautical miles from the shore (Fig 9). All trawlers observed in Pondicherry were in violation of the minimum mesh size allowed along with 20% of the gill nets. Additionally, all ring seines observed in Pondicherry were in complete violation according to the Pondicherry Fishing Regulation Act where it clearly states that ring seines are banned (Fig 10).

**Table 8:** Marine Fishing Rules and Regulations for Tamil Nadu (1983) and Pondicherry (2009). Gear mesh regulations differed between Tamil Nadu and Pondicherry and the respective region has been indicated in brackets. NA (Not applicable) indicates the rule does not apply in that region. Trawlers were considered cumulatively and were not differentiated into prawn and fish trawlers.

Marine Fishing Rules and Regulations	Violations (% of total observations)	
	Tamil Nadu 1983	Pondicherry 2009
Rule 1: Only if fishing beyond three nautical miles: Mechanised craft must leave at 5 AM and return by 9 PM only to leave at 5 AM next day.		
Multi day fishing beyond the three nautical mile limit	7	66
Multi day fishing within the three nautical mile limit	3	13
Rule 2: Mechanised craft must not fish within three nautical miles from the shore		
Mechanised craft (trawlers + vallams) within three nautical miles	68	15
Trawlers within three nautical miles	93	15
Vallams within three nautical miles	20	17
Rule 3: Specific gear mesh size regulations		
No fishing gear less than 10 mm. mesh from knot to knot in respect of nets other than trawl net shall be used (Tamil Nadu regulation)*	0	NA
No gill net having a stretched mesh size less than 25 mm from knot to knot shall be used (Pondicherry regulation)**	NA	20
No shrimp trawl net having a stretched mesh size less than 37 mm. at the cod-end shall be used (Pondicherry regulation)**	NA	100
No fish trawl nets having a stretched mesh size less than 75 mm. at the wings and 40 mm. at cod-end shall be used (Pondicherry regulation)**	NA	100

## Discussion

Our study maps the spatial extent of small scale fisheries along two districts of Tamil Nadu and the Union Territory of Pondicherry along the Coromandel Coast for the first time. It also examines the spatial aspect and application of fishing regulations for each respective region. Key findings of the study are distinct high pressure fishing zones, overlapping fishing territories between traditional, motorised and mechanised craft and large scale violations of existing regulations along the Coromandel Coast.

Visualisations of the extent and intensity of fishing in addition to identification of violations of fisheries laws are extremely important to be able to identify appropriate management approaches particularly within complex small scale fisheries [16]. The small scale fisheries community particularly in this region is rife with conflict mostly because of high competition of resources and resulting severe economic disparities. India’s fishing regulations do not, at this point, set any quota or restrictions on the amount of catch that a given craft may capture. Thus, as illustrated in Table 9, there is a significant difference between the catch extracted by the trawlers as they have a higher capacity than traditional or motorised craft which points to an increasing income disparity within the community [75]. A major outcome of this study alludes to this continued conflict specifically over territory leading to resource and therefore economic and social conflicts that impacts resource management substantially [76].

**Table 9:** Catch and Earnings available per fishing day per crew member categorised by the different types of gear types employed along the Coromandel Coast. Trawlers and ring seines clearly capture larger volumes in comparison to the gill nets, shore seines and lines. Trawl fishermen also earn the highest amount while the traditional fishermen earn the least. Table adapted from Lawrence, 2016 [75].

Type of Gear	No. of samples	Mean Mesh Size (mm)	Mean Crew size	Catch per day per person (kg)		Earnings per day per person (INR)		Catch weight (kg)	
				Mean	SE	Mean	SE	Mean	SE
Lift net	7	24	33	34.87	7.73	128.12	68.47	1300.00	141.42
Line	119	--	4	10.09	2.15	498.71	796.52	43.87	12.73
Mono-filament Gill Net	3646	46	3	6.07	0.1	208.7	181.78	16.52	0.33
Multi-filament Gill Net	365	43	3	5.34	0.33	286.32	566.78	12.48	0.89
Ring seine	61	23	34	35.11	3.45	361.26	231.08	1135.57	110.44
Scoop net	12	24	4	22.88	2.97	689.58	204.9	91.67	15.13
Shore seine	19	60	22	4.09	1.1	86.43	110.43	92.89	25.44
Trawl net	435	20	5	43.06	3.28	2397.7	1407.46	385.20	28.98

Historically, traditional or artisanal fisheries in India have been restricted to fishing within depths of 50 m and within three nautical miles, particularly in Tamil Nadu [52]. The modernisation of the traditional fleet began as early as the 1950s where large scale motorisation of traditional craft occurred alongside a major shift towards mechanised fishing or trawling [51]. This altered the social and economic structure of fishing communities considerably in the pursuit for prawns or “pink gold”, which as the term suggests, were considered extremely valuable [55, 59].

The resulting explosion of fishing effort in inshore waters was unexpected as trawlers began fishing in the same areas as artisanal fishermen leading to conflict between the traditional and mechanised fishing sectors [53]. This also resulted in a massive increase in fishing effort within an inshore zone of approximately 10 km from the coast and 30 m depth. Inshore fisheries resources were indiscriminately exploited and all available gear was used. This led to a fundamental change in the catch composition. Presently the catch is dominated by a few lower trophic level species, lower abundances and smaller sizes [75].

Both traditional and motorised sectors continue to adopt fishing methods that maximise catch, however unsustainable, via the use of smaller mesh sizes, illegal and/or modified gear [77]. This is expected, given limited stocks and high competition. For example, the use of ring seines which are illegal, indicates an increased dependence of the community on oil sardines and other small pelagic fish stocks which typically exhibit boom and bust population dynamics [78]. A collapse of these stocks has major ecological and economic implications for other commercial fisheries they support and should therefore be managed rather than exploited indiscriminately [79]. Another negative aspect of these gears is the huge capital involved which pushes fishermen further into debt via informal credit arrangements which are common in developing countries [80]. Even though ring seine catches appear to be more lucrative than catches from gill nets and shore seines, pelagic stocks may not survive the existing fishing capacity which in turn could result in large scale debt within the community. In parallel is another emerging trend where “modified trawls” were used such that the net is modified to work like a trawl net but is smaller and is operated by two smaller motorised or traditional craft. As trawlers actively target by-catch species [81], it is alarming that the smaller boats have also resorted to trawling further inshore for the same (Table 3).

Overlapping fishing zones by different fishing gear and craft coupled with the ubiquitous use of unsustainable fishing methods call for an urgent need for fisheries management, before all inshore stocks collapse. In this context, maps visualising the different combinations of gear and craft in terms of fishing density or pressure are a major outcome of this study. These maps can serve as a tool for spatially explicit management approaches such as periodic area closures [82], no fishing zones or marine protected areas [32, 42, 83, 84].

The marine fisheries regulations for Pondicherry and Tamil Nadu clearly specify that the mechanised fleet are to fish only beyond three nautical miles as the area within this limit is reserved for traditional and motorised craft [85, 86]. Our study clearly demonstrates an unfortunate yet wide disconnect between legislation and ground reality. Even specified classifications of mechanised and deep sea fishing vessels widely differ from active fishing vessels in coastal waters. The three nautical mile limit and the multi day fishing regulation is largely ignored by mechanised boats all along the coast of Tamil Nadu and Pondicherry. Mesh size regulations are extremely liberal in Tamil Nadu such that 10 mm is regarded as the minimum legal limit for all gears except trawlers. As a result, no violations of this mesh limit was recorded in Tamil Nadu yet the ecological implications of such a low limit are fairly evident and are of concern. Conversely, the relatively more recent regulations for Pondicherry had more stringent specifications for mesh sizes used by gill nets and trawlers in addition to clearly declaring the ring seine as an illegal gear. Violations of these regulations also point to major problems with fisheries legislation for this region.

Notably, existing legislation was originally conceived to address the rising conflict between the traditional fishermen and trawlers following the introduction of trawling, and not management of fishery resources [60]. Perhaps violations of the same could then be attributed to the lack of awareness within the fishing community or the fact that different rules apply to two states that share the coastline and adjacent waters and where fishermen from one state can and do land their catch in the other state. Nevertheless, the implementation and subsequent enforcement of existing legislation has been a glaring failure and remains a significant challenge [59]. If current legislation were actually enforced, particularly the three nautical mile rule along with the mesh regulations, the issue of territorial conflict will be addressed and some form of management would exist.

The Coromandel Coast has a long history of traditional fisheries management led by village councils or panchayats that decided larger issues like potential new gears or new locations to fish [54]. However, such practices were considered unscientific and were therefore largely neglected by the Government. Instead, trawlers enjoyed unfettered support, which eventually led to structural collapses of panchayats or village councils in several villages [53, 54]. Ironically, village councils where extant are still regarded by Government Fisheries officials and are frequently consulted on issues related to fisheries management. Notably, the existence of fishermen laws such as the local ring seine ban of six months [75] or the ban on the scoop net (kacchavalai) [54] that are enforced by the community, clearly demonstrates the strength and potential role of the community in fisheries management. Even though these laws are limited in scope, the fact that they are followed indicates the willingness of the community to adhere to community based management mechanisms rather than State law and herein lies an opportunity. If governments were to shift their focus from supporting trawlers and encouraging maximum exploitation towards strengthening the role of the community for fisheries management, there would be a more positive dialogue which could lead to the beginning of a working collaboration for management [75]. Indeed, fishermen and village leaders had several specific recommendations for action when we shared the results of our study and held interactive discussions encouraging their thoughts on action needed for management of local stocks. Their specific recommendations are as below:

1. Reconciling the two MFRAs of Pondicherry and Tamil Nadu to remove contradictions and strengthening of the respective fisheries departments so they can participate more actively in fisheries management and enforcement.
2. Enforcement of the three nautical mile limit for mechanised craft. (This recommendation was vociferously supported by artisanal fishers.)
3. Review and revision of mesh size regulations to make them specific to types of nets and targeted species. Two specific recommendations to facilitate this were:
  - Buy back of nets that do not conform to mesh size regulations.
  - Enforce a ban on the manufacture and sale of these nets so that their production and sale to fisherfolk is prevented at source.
4. Creation of no-fishing zones along the coastline to provide refuge and nurseries for fish.

Despite the enthusiasm of being involved in the process and understanding the need for management, the common consensus was that the bigger more powerful villages needed to be on board. These bigger villages tended to be comprised of trawl fishermen who had considerable political influence. Indeed, strong political will is a major factor in the success of any management initiative as suggested by several examples of small scale fisheries in Asia [8, 87]. Nevertheless, it is clear that a combination of factors that provide for an integrated and adaptive approach to management is required, primarily with the community in the forefront with further support from the Government and other key stakeholders [6, 88, 89]. Our study clearly demonstrates the role fishermen can play in research and subsequently, management and should therefore be recognised with optimism as there is potential for a community based management approach to work provided a few fundamental changes in organisation and prevailing perceptions take place.

## Supporting information

S1 Fig Fishing craft found along the Coromandel coast of Tamil Nadu. (a) A traditional Kattumaram, (b) a Kattumaram fitted with an outboard engine, (c) a small and (d) large fibre reinforced plastic boats. (e) trawler, (f) Vallam.
(TIF)

S1 Table Description of attributes in the GIS layer and CSV file. (CSV)

S1 File Code and code-documentation with datasets. (ZIP)

S2 File Observations of craft as point locations with attributes collected during boat and crew surveys and shortest distance to the shoreline. (CSV)

S3 File Interactive map of kernel density estimates and location of type of gear and mesh sizes of gear they operate. (HTML)

S4 File Interactive map of kernel density estimates and location of craft type and mesh sizes of gear they operate. (HTML)

S5 File Interactive map of kernel density estimates and location of all craft and mesh size classes of gear they operate. (HTML)

S6 File Interactive map of kernel density estimates and location of different craft classes and mesh sizes of gear they operate. (HTML)
