# Supplementary figures and images for: Spatially explicit action research for coastal fisheries management
Source: PLoS One. 2018 Jul 11;13(7):e0199841. doi: 10.1371/journal.pone.0199841 (PMC6040741; doi:10.1371/journal.pone.0199841)

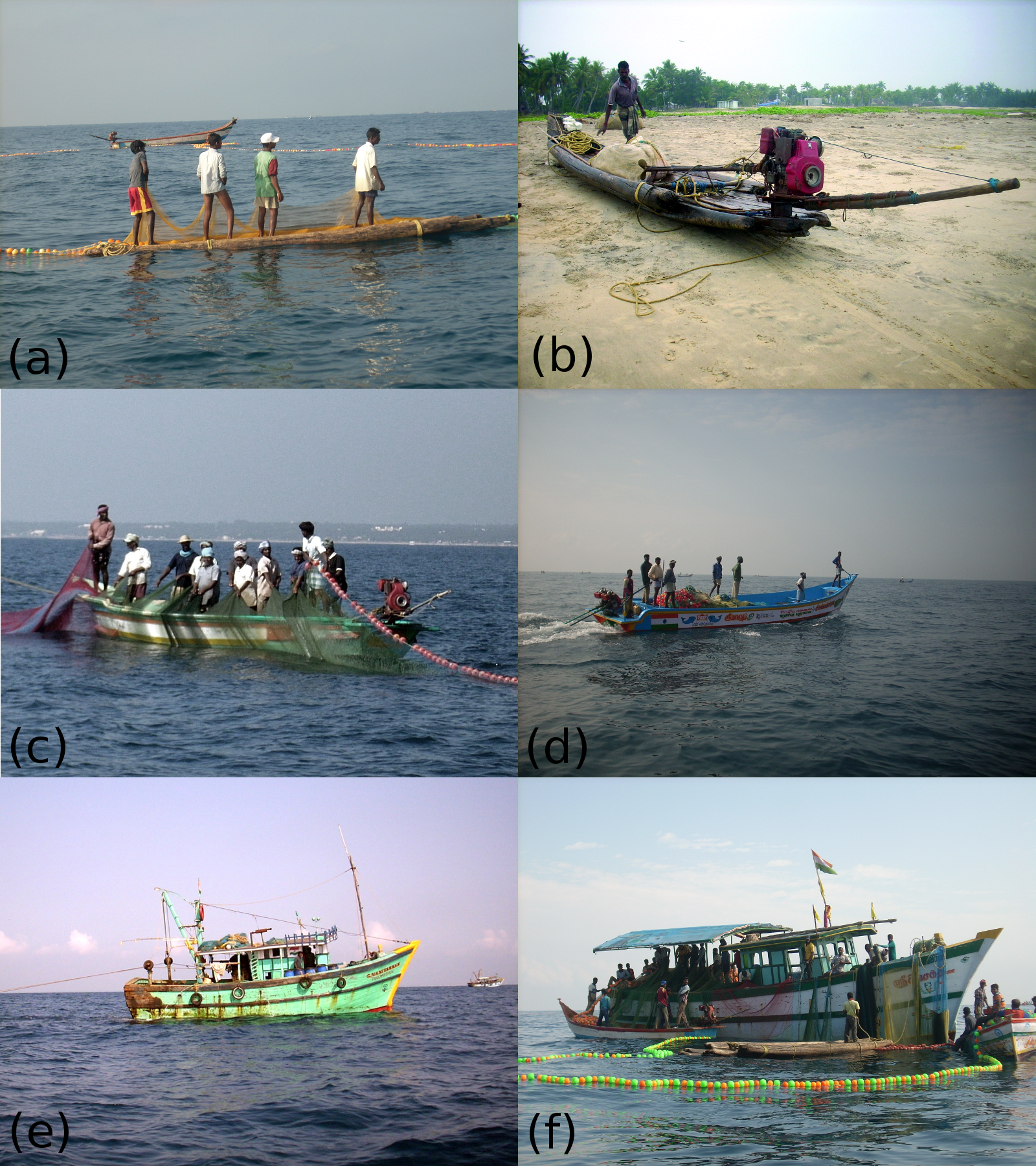

Supplement: S1 Fig — (a) A traditional Kattumaram, (b) a Kattumaram fitted with an outboard engine, (c) a small and (d) large fibre reinforced plastic boats. (e) trawler, (f) Vallam. (TIF) [file pone.0199841.s001.tif]
