# Supplementary material for: Spatially explicit action research for coastal fisheries management
Source: PLoS One. 2018 Jul 11;13(7):e0199841. doi: 10.1371/journal.pone.0199841 (PMC6040741; doi:10.1371/journal.pone.0199841)
Supplement: S1 File — (ZIP) [file pone.0199841.s003.zip › CodeDocumentation.html]

Code documentation for paper "Spatially Explicit Action Research for Coastal Fisheries Management"


# Code documentation for paper "Spatially Explicit Action Research for Coastal Fisheries Management"

## Tara Lawrence and R.S. Bhalla

## Table of Contents

- Summary
- Initialise
  - Set up the environment
  - Load the libraries
  - Datasets
  - Functions and initialisation scripts
- Run the non-parametric statistics
  - Summarise the data
  - Plot to see the distribution of the data
  - Plotting box plots to show differences between fisheries and craft types
  - Kruskal Wallis for non parametric
- SPATIAL ANALYSIS
  - Spatial patterns
  - Spatial patterns for mesh size distributions
  - plotting distance violations
- Generate plots
  - generate histogram of distance from shoreline
- Mapping
  - Get the basemaps and panels
- GIS data processing using GRASS and R
  - Create a 10km grid over the data and clean up legacy files
  - Create subsets of maps
  - Process craft classes
  - Build quadrats around fisheries
  - Build quadrats around mesh sizes
- Generate interactive maps
  - Craft and mesh size
  - Mesh classes and their distribution
  - Net types and mesh sizes
  - Craft classes and mesh sizes

## Summary

This is supplementary material to allow interested persons to replicate the results of the data. This is packaged along with the code and the dataset. The code blocks embedded within this document has been extracted (tangled) into the file `FisheriesMappingScript.R`. You need to be connected to the internet for the code to work as the OSM maps are yanked off the web. The script was created using Emacs 2.5 with ESS and org-babel.

Details of the system and R version used are:

```
R.Version()
```

| platform | arch | os | system | status | major | minor | year | month | day | svn.rev | language | version.string | nickname |
| --- | --- | --- | --- | --- | --- | --- | --- | --- | --- | --- | --- | --- | --- |
| x86\_64-pc-linux-gnu | x86\_64 | linux-gnu | x86\_64, linux-gnu |  | 3 | 4.4 | 2018 | 3 | 15 | 74408 | R | R version 3.4.4 (2018-03-15) | Someone to Lean On |

## Initialise

This will set up the environment, unzip and create the data folder and load the required libraries.

NOTE
:   You should be running this script from within the folder you intend to copy the data to.

### Set up the environment

```
setwd("./")
print(paste("The working directory is:", getwd()))
```

```
[1] "The working directory is: /home/udumbu/rsb/Papers/Fisheries/Mapping/PLOS/rscript"
```

### Load the libraries

In case the output here throws an error, you have a library missing or, the library is outdated. Please use the command `install.package("NameOfPackage", dependencies=TRUE)` to install or update the missing packages.

```
library(tools)
library(spatstat) # spatial point pattern analysis
library(spdep) # spatial statistics
library(raster) # raster operations
library(rgeos) # import export of geographical data
library(ggplot2) # graphing and visualising
library(ggmap) # mapping in ggplot
library(ggsn) # map furniture
library(sp)
library(grid) # manipulate ggplot2 objects
library(gtable)# manipulate ggplot2 objects
library(gridExtra) # graphing and visualising
library(ggExtra) # graphing and visualising
library(lattice) # graphing and visualising
library(maptools) # tools to manipulate geographical data
library(rgdal) # manipulate raster images
library(rgl) # 3-D visualisation
library(lmtest)
library(pgirmess) # kruskal wallis multi component analysis; load pgirmess library
library(extrafont) #load fonts for Plos One
library(rgrass7)
library(gstat)
library(doBy) # summary stats
library(dunn.test) #post hoc tests after Kruskal Wallis
library(plotly) # generate interactive maps
```

| x |
| --- |
| plotly |
| dunn.test |
| doBy |
| gstat |
| rgrass7 |
| XML |
| extrafont |
| pgirmess |
| lmtest |
| zoo |
| rgl |
| rgdal |
| maptools |
| lattice |
| ggExtra |
| gridExtra |
| gtable |
| grid |
| ggsn |
| ggmap |
| ggplot2 |
| rgeos |
| raster |
| spdep |
| spData |
| Matrix |
| sp |
| spatstat |
| rpart |
| nlme |
| spatstat.data |
| tools |
| stats |
| graphics |
| grDevices |
| utils |
| datasets |
| methods |
| base |

### Datasets

Check to see if datasets exist, else unzip the data file. Then create a folder to hold the results. Note the datasets include a data directory with the ESRI shapefiles and a GRASS database.

```
if(!dir.exists("./Data"))unzip("Datasets.zip", exdir = "./Data")
if(!dir.exists("./grassdata"))unzip("grassdata.zip", exdir = "./grassdata")
dir.create("./Results")
```

```
Warning message:
In dir.create("./Results") : './Results' already exists
```

Read in the data

```
fc.data <- readOGR(dsn = "./Data", "BtDistUTM", integer64 = "warn.loss", stringsAsFactors = FALSE) # data is in UTM 44North EPSG 32644
fc.data$mesh1mm <- as.numeric(fc.data$mesh1mm) ## NAs are for line and hook
fdf <- as.data.frame(fc.data) # ggplot only works on dataframes
spatial.df<-read.csv("./Data/spatial2017.csv", stringsAsFactors=FALSE) # cleaned data for npstat.R
```

```
OGR data source with driver: ESRI Shapefile 
Source: "/home/udumbu/rsb/Papers/Fisheries/Mapping/PLOS/rscript/Data", layer: "BtDistUTM"
with 3427 features
It has 26 fields
Integer64 fields read as signed 32-bit integers:  ID slno crew_size no_boats1 no_boats2 tot_boats boat_lengt survey_mon no_days
Warning message:
NAs introduced by coercion
```

### Functions and initialisation scripts

Some functions to assist with the plotting. Note that code has been borrowed from various sources, mostly stackexchange.

- Extract legend  

  Shares a legend between two ggplot graphs. Source: https://github.com/hadley/ggplot2/wiki/Share-a-legend-between-two-ggplot2-graphs

  ```
  g_legend<-function(a.gplot){
  tmp <- ggplot_gtable(ggplot_build(a.gplot))
  leg <- which(sapply(tmp$grobs, function(x) x$name) == "guide-box")
  legend <- tmp$grobs[[leg]]
  return(legend)
  }
  ```
- Write both eps and tiff  

  ```
  eps.tiff <- function(plot.cmd, flnm, width.in, height.in){
    tiff(filename = paste0(flnm, ".tiff"),
      width = width.in, height = height.in,
      units = "in", compression = "lzw", pointsize = 12, res = 450, family = "Arial",
      type = "cairo", antialias="subpixel")
    plot(plot.cmd)
    dev.off()
    cairo_ps(filename = paste0(flnm, ".eps"),
      width = width.in, height = height.in, pointsize = 12,
      onefile = FALSE, family = "Arial", bg = "white",
      antialias = "subpixel", fallback_resolution =450)
    plot(plot.cmd)
  dev.off()
  }
  ```
- Create weight files for tests of auto-correlation  

  ```
  get.weights <- function(long, lat){
      xy <- cbind(long, lat)
      map_nb <<- knn2nb(knearneigh(xy, longlat=TRUE))
      map_nb <<- make.sym.nb(map_nb)
      map_w <<- nb2listw(map_nb,glist=NULL,style="W",zero.policy=FALSE)
      map_b <<- nb2listw(map_nb, glist=NULL, style="B", zero.policy=FALSE)
  }
  ```
- Run the local Geary's test and output the data in a formatted table  

  ```
  gry.test <- function(x){
    df <- as.data.frame(matrix(nrow=1, ncol=5))
    names(df) <- c("data.name", "Geary C statistic", "Expectation", "Variance", "p.value")
    gt <- geary.test(x, map_w, randomisation=TRUE, zero.policy=NULL, alternative="greater", spChk=NULL, adjust.n=TRUE)
    df[1,1] <- gt$data.name
    df[1,2:4] <- gt$estimate
    df[1,5] <- gt$p.value
    write.table(df,"./Results/GearyTest2.csv", append=TRUE, row.names=TRUE)
    return(df)
  }
  ```
- Initialise GRASS  

  Ensure you're pointing this to the correct folder (check your installed GRASS version) do this only when running the script outside GRASS. Else you need to run R from within GRASS. Type `?rgrass7` for more information.

  ```
  initGRASS(gisBase = "/usr/lib/grass74/", home = tempdir(), 
    gisDbase = "./grassdata",
    location = "fisheries", mapset = "craftdist", override = TRUE)
  ```

  ```
  gisdbase    ./grassdata 
  location    fisheries 
  mapset      craftdist 
  rows        162 
  columns     111 
  north       1407232 
  south       1245267 
  west        365066.1 
  east        476022 
  nsres       999.7841 
  ewres       999.603 
  projection  +proj=utm +no_defs +zone=44 +a=6378137 +rf=298.257223563
  +towgs84=0.000,0.000,0.000 +to_meter=1
  ```
- Plot Panels  

  ```
  plot.panels <- function(panels){
  craft.map <- ggmap(pdymap.bw,
                     base_layer = ggplot(aes(x = long, y = lat), data = fdf))  +
      geom_point(shape = 1) +
      labs(x = 'Longitude', y = 'Latitude') +
      facet_wrap(as.formula(paste("~", panels)))
      ggsave(filename = paste0(panels, ".png"))
      }
  ```
- Multi-plot panels  

  ```
  qmplot.panels <- function(panels){
  craft.map <- qmplot(long, lat, data = fdf, maptype = "toner-lite", extent = "panel")  +
      geom_point(shape = 1) +
      theme(plot.caption = element_text(colour="black", size=6, face="bold"),
      axis.text = element_text(colour="black", size=7)) +
      labs(x = 'Longitude', y = 'Latitude', caption = "NOT TO SCALE") +
      facet_wrap(as.formula(paste("~", panels)))
  ggsave(filename = paste0("./Results/PlotPanel", panels, ".png"))
  print(craft.map)
  }
  ```
- Create new GRASS layers based on a list of input files through sub-setting  

  Creates new layers based on input list by subsetting maps through v.extract. Use as lapply(X = map.list, FUN = subset.map, colhd). Here `in.list` is the filter criteria for extracting data; `colhd` is the field in which these filter criteria lie. For example for the colhd = fishery, the list is ("trawl", "gillnet"….)

  ```
  subset.map <- function(map.list, colhd){
  map.out <- gsub(" ", "", map.list, fixed = TRUE) #remove whitespace
  map.out <- paste0(map.out,"_",colhd) # append col name to output map
  execGRASS("v.extract", input="BtDistUTM@PERMANENT", type="point",
            where = paste0(colhd, " = \'", map.list,"\'"),
            output=map.out, flags = "overwrite", intern = TRUE)
            }
  ```
- Subset mesh size  

  Input is size range as `lwr` (lower boundary) `upr` (upper boundary) and `nm` (name) of output. Use as mapply(FUN, lwr=, upr=, nm=)

  ```
  subsetmesh <- function(lwr, upr, nm){
  query <- paste("meshsize >=", lwr, "and meshsize <", upr)
  execGRASS("v.extract", input="BtDistUTM@PERMANENT",
  type="point", where=query,
            output=nm, flags = "overwrite")
            }
  ```
- Generate quadrat  

  The function generates a quadrat of 1km, sum number of boats and puts the data to the centroid of that quadrat for subsequent visualisation. Use with mapply with x = net.maps, y = net.ptmaps.

  ```
  build.quads <- function(x, y){
  execGRASS("v.select", parameters = list (ainput="onekmgrid@craftdist", atype="area", binput=x, btype=c("point","area"), output="quad1k@craftdist", operator="contains"), flags = "overwrite") # select only those grids with boats
  ## see if a table exists
  if(identical(execGRASS("v.db.connect", flags = "c", map = x, intern=TRUE), character(0)))
  execGRASS("v.db.addtable", map=x, columns="tot_boats INT") # create table and add col
  ## execGRASS("v.db.addcolumn", map=x, columns="tot_boats INT") #add column if table exists
  if(execGRASS("v.info", map=x, flags = "t", intern = TRUE)[2] != "points=0"){
  execGRASS("v.vect.stats", parameters = list(points=x, areas="quad1k@craftdist", count_column="count", method="sum", points_column="tot_boats", stats_column="sum_bts"))
  ## count the points in each grid
      execGRASS("v.extract", input="quad1k@craftdist", type="centroid", output="quad1kcent@craftdist", flags = "overwrite")
      execGRASS("v.type", input="quad1kcent@craftdist", output=y, from_type="centroid", to_type="point", flags = "overwrite") # generate a centroid map
      }else{
      print("layer is empty")
      }
      }
  ```
- Create a data frame of IDW interpolated predictors  

  Create a data frame of IDW interpolated predictors for each of the fisheries and `rbind` into a single data frame for plotting. Use with mapply and rbind with x = net.ptmaps, y = fishery.maps

  ```
  run.idw <- function(x, y){
  boatdens <- readVECT(x, plugin=NULL)
  bt.ll <- spTransform(boatdens,  CRS("+proj=longlat +datum=WGS84")) # change crs to lat long
  ## bt.ll$efrt <- bt.ll$sum_bts
  ## bt.pp <- as.ppp(bt.ll)
  grd <- as.data.frame(spsample(bt.ll, "regular", n=100000))
  names(grd) <- c("x", "y")
  coordinates(grd) <- c("x", "y")
  gridded(grd) <- TRUE  # Create SpatialPixel object
  fullgrid(grd) <- TRUE  # Create SpatialGrid object
  proj4string(grd) <- CRS(proj4string(bt.ll)) 
  ## Interpolate the surface using a power value of 2 (idp=2.0)
  dat.idw <- gstat::idw(sum_bts~1,bt.ll,newdata=grd,idp=2.0)
  idw.df <- as.data.frame(dat.idw)
  idw.df$facet <- y
  return(idw.df)
  }
  ```
- Plot the quadrat count map  

  Function to ggplot the map. Input is ggdat created from the call above.

  ```
  plot.quadcount <- function(plot.data){
  ggdat <<- plot.data
  plot <- ggmap(pdymap.bw, base_layer = ggplot(aes(x = x, y = y, z = var1.pred), data=ggdat)) +
  facet_wrap( ~ facet, ncol = 3) +
  geom_raster(aes(alpha=var1.pred))+    
      labs(alpha = "Boats/km2", x = "long", y = "lat") +
      geom_contour(alpha = .25) +
      coord_equal()
      return(plot)
      }
  ```
- Multi-plot the quadrat count map  

  ```
  qmplot.quadcount <- function(ggdat){
  plot <- qmplot(x = x, y = y, data = ggdat, maptype = "toner-lite", extent = "panel", alpha = I(.0)) +
      theme(plot.caption = element_text(colour="black", size=6, face="bold")) +
      theme(axis.text = element_text(colour="black", size=7)) +
      labs(alpha = "Boats/km2", x = 'Longitude', y = 'Latitude', caption = "NOT TO SCALE") +
      facet_wrap( ~ facet) +
      geom_raster(aes(alpha=var1.pred))+
      geom_contour(alpha = .25, aes(x = x, y = y, z = var1.pred)) +
      coord_equal()
      return(plot)
      }
  ```
- Read a list of vector files  

  ```
  read.vect.list <- function(x, facet.x){
  vect.x <- readVECT(x)
  df.x <- as.data.frame(vect.x)    
  df.x$facet <- facet.x
  return(df.x)
  }
  ```
- Multi-plot a 2-d kernel density dataset  

  ```
  qmplot.dens2d.mesh <- function(ggdat, fig.filename){
  out <- by(data = ggdat, INDICES = ggdat$facet, FUN = function(m) {
      m <- droplevels(m)
      qmplot(x = long, y = lat, data = ggdat, maptype = "toner-lite", extent = "panel", zoom=10, geom="text", label="") + # force extents to fit data locations #qmplot
          geom_point(data = m, aes(x = long, y = lat, colour=meshsize)) +
          stat_density2d(data = m, aes(x = long, y = lat)) +
          labs(
              x = NULL,
              y = NULL ,
              title = m$facet[1],
              colour = "Mesh \nSize (mm)",
              caption = NULL # "Not to scale."
          ) +
          coord_equal()
  })
  grb.out <- do.call(arrangeGrob, c(out, list(ncol=3, padding = 0)))
  x.lab <- textGrob("Longitude", gp=gpar(fontsize=14))
  y.lab <- textGrob("Latitude", rot=90,gp=gpar(fontsize=14))
  grid.arrange(grb.out, left=y.lab, bottom=x.lab, widths=c(100, 0.5, 0.0))

  if(file_ext(fig.filename)=="tiff"){
      ## fig.file <-
      tiff(filename = fig.filename, width = 12, height = 9,
           units = "in", compression = "lzw", pointsize = 12, res = 300, family = "Arial",
           type = "cairo", antialias="subpixel")
      grid.arrange(grb.out, left=y.lab, bottom=x.lab, widths=c(100, 0.5, 0.0))
      dev.off()
  }else{
      cairo_ps(filename = fig.filename, width = 12, height = 9, pointsize = 12,
               onefile = FALSE, family = "Arial", bg = "white",
               antialias = "subpixel", fallback_resolution =600)
      grid.arrange(grb.out, left=y.lab, bottom=x.lab, widths=c(100, 0.5, 0.0))
      dev.off()
    }
  }
  ```

## Run the non-parametric statistics

### Summarise the data

#### Summary of mesh sizes by type of net

```
summaryBy(mesh1mm~fishery,data = spatial.df, FUN=c(median, sd, mean), na.rm=TRUE)
```

```
    fishery mesh1mm.median mesh1mm.sd mesh1mm.mean
1   Gillnet             46  16.642336    46.281911
2      Lift             12   0.000000    12.000000
3      Line              6   3.953912     9.115942
4 Ringseine             28  37.736702    51.384977
5  Scoopnet             12   0.860663    12.074074
6     Trawl             20   1.387490    20.064257
```

#### Summary of depth of fishing by type of net

```
summaryBy(depth_m~fishery,data = spatial.df, FUN=c(median, sd, mean), na.rm=TRUE)
```

```
    fishery depth_m.median depth_m.sd depth_m.mean
1   Gillnet          12.25   5.188013    13.397689
2      Lift          14.08   2.485833    14.355000
3      Line          24.51  21.624134    28.640435
4 Ringseine          24.69  21.499570    30.607786
5  Scoopnet           8.05   2.370998     7.828963
6     Trawl          24.51  15.991006    27.336233
```

#### Summary of distance to shore by net

```
summaryBy(shoredist_km~fishery,data = spatial.df, FUN=c(median, sd, mean), na.rm=TRUE)
```

```
    fishery shoredist_km.median shoredist_km.sd shoredist_km.mean
1   Gillnet                1.82        2.430419          2.697331
2      Lift                4.11        1.611076          3.955000
3      Line               13.26       11.214906         12.904348
4 Ringseine               10.30        8.268917         10.737324
5  Scoopnet                0.80        1.521430          1.074370
6     Trawl               10.88        8.450241         10.823719
```

#### Summary of mesh sizes by craft type

```
summaryBy(mesh1mm~boat_type1,data = spatial.df, FUN=c(median, sd, mean), na.rm=TRUE)
```

```
  boat_type1 mesh1mm.median mesh1mm.sd mesh1mm.mean
1        frp             52  20.714772     41.03734
2    frp_big             75  25.859127     76.63889
3 kattumaram             44  12.742809     43.31197
4      trawl             20   2.627919     19.57377
5     vallam             34  38.841742     53.41304
```

#### Summary of depth of fishing by craft type

```
summaryBy(depth_m~boat_type1,data = spatial.df, FUN=c(median, sd, mean), na.rm=TRUE)
```

```
  boat_type1 depth_m.median depth_m.sd depth_m.mean
1        frp          13.72   5.674216     14.40787
2    frp_big          16.09   4.338676     16.57528
3 kattumaram           9.69   2.802077     10.15599
4      trawl          25.24  16.173801     28.72341
5     vallam          26.70  21.598749     33.53738
```

#### Summary of distance to shore by craft type

```
summaryBy(shoredist_km~boat_type1,data = spatial.df, FUN=c(median, sd, mean), na.rm=TRUE)
```

```
  boat_type1 shoredist_km.median shoredist_km.sd shoredist_km.mean
1        frp               2.585        2.644221          3.288047
2    frp_big               3.975        1.677750          4.062222
3 kattumaram               0.880        1.063816          1.213758
4      trawl              11.355        8.509531         11.660279
5     vallam              11.450        8.086057         12.068750
```

### Plot to see the distribution of the data

#### Overall distribution via frequency curves/histograms and scatterplots

```
p <- ggplot(spatial.df, aes(depth_m, shoredist_km)) + geom_point() + theme_classic()
ggExtra::ggMarginal(p, type = "histogram")
```

Data are strongly positive skewed

#### Plotting variables to visualise skew

- Shoreline distance  

  ```
  ggplot(spatial.df, aes(shoredist_km)) + geom_freqpoly(bins = 20)
  ```
- Depth  

  ```
  ggplot(spatial.df, aes(depth_m)) + geom_histogram(bins = 20)
  ```
- Mesh size  

  ```
  ggplot(spatial.df, aes(mesh1mm)) + geom_freqpoly(bins = 10)
  ```

  Data is non-normal, strong positive skew, we need to use non-parametric tests.
- Distribution of fishing points by distance and bottom depth  

  ```
  ggplot(spatial.df, aes(shoredist_km, depth_m)) + geom_point()
  ```

### Plotting box plots to show differences between fisheries and craft types

#### Plotting for bottom depth by fishery (net type)

```
ggplot(spatial.df,aes(fishery, depth_m)) + stat_boxplot(geom ='errorbar') + geom_boxplot(notch = TRUE) #, outlier.shape=NA)
p<-ggplot(spatial.df,aes(fishery, shoredist_km)) + stat_boxplot(geom ='errorbar') + geom_boxplot(notch = TRUE, coef = 1.5, outlier.shape=NA) + scale_y_continuous(limits = c(0, 38))
b<- p + xlab("Fishery") + ylab("Distance to shore (km)") + ggtitle("Distance to Shore") 
c<-b + theme(axis.title.x = element_blank(), axis.title.y = element_text(face="plain", size=12))
d<-c + theme(axis.text = element_text(size=10, face="plain"))
e<-d + theme(plot.title = element_text(size = 12, face = "plain", hjust = 0.5))
e
```

#### Plotting for bottom depth by fishery (net type)

```
f<-ggplot(spatial.df,aes(fishery, depth_m)) + stat_boxplot(geom ='errorbar') + geom_boxplot(notch = TRUE, coef = 1.5, outlier.shape=NA) + scale_y_continuous(limits = c(0, 70))
g<- f + xlab("Fishery") + ylab("Bottom depth (m)") + ggtitle("Depth of Operation") 
h<-g + theme(axis.title.x = element_blank(), axis.title.y = element_text(face="plain", size=12))
i<-h + theme(axis.text = element_text(size=10, face="plain"))
j<-i + theme(plot.title = element_text(size = 12, face = "plain", hjust = 0.5))
j
```

Plots are very similar suggesting the obvious correlation between depth and distance from shore. Put the two plots together and save as eps and tiff.

```
plot.cmd <- grid.arrange(e, j,  layout_matrix = matrix(c(1,2), ncol=2, byrow=TRUE))
flnm <- "./Results/FishingDistancesDepth_byGear"
eps.tiff(plot.cmd, flnm, width.in = 12, height.in = 6)
plot.cmd
```

+

#### Plotting for bottom depth and distance of fishing by craft type

```
ggplot(spatial.df,aes(boat_type1, depth_m)) + stat_boxplot(geom ='errorbar') + geom_boxplot(notch = TRUE)  #, outlier.shape=NA)
p<-ggplot(spatial.df,aes(boat_type1, shoredist_km)) + stat_boxplot(geom ='errorbar') + geom_boxplot(notch = TRUE, coef = 1.5, outlier.shape=NA) + scale_y_continuous(limits = c(0, 35))
p
b<- p + xlab("Type of fishing craft") + ylab("Distance to shore (km)") + ggtitle("Distance to Shore") + scale_x_discrete(breaks=c("frp", "frp_big", "kattumaram","trawl","vallam"), labels=c("Small FRP", "Big FRP", "Kattumaram", "Trawler", "Vallam"))
c<-b + theme(axis.title.x = element_blank(), axis.title.y = element_text(face="plain", size=12))
d<-c + theme(axis.text = element_text(size=10, face="plain"))
e<-d + theme(plot.title = element_text(size = 12, face = "plain", hjust = 0.5))
e

f<-ggplot(spatial.df,aes(boat_type1, depth_m)) + stat_boxplot(geom ='errorbar') + geom_boxplot(notch = TRUE, coef = 1.5, outlier.shape=NA) + scale_y_continuous(limits = c(0, 65))
f
g<- f + xlab("Type of fishing craft") + ylab("Bottom depth (m)") + ggtitle("Depth of Operation") + scale_x_discrete(breaks=c("frp", "frp_big", "kattumaram","trawl","vallam"), labels=c("Small FRP", "Big FRP", "Kattumaram", "Trawler", "Vallam"))
h<-g + theme(axis.title.x = element_blank(), axis.title.y = element_text(face="plain", size=12))
i<-h + theme(axis.text = element_text(size=10, face="plain"))
j<-i + theme(plot.title = element_text(size = 12, face = "plain", hjust = 0.5))
j

plot.cmd <- grid.arrange(e, j, layout_matrix = matrix(c(1,2), ncol=2, byrow=TRUE))
flnm <- "./Results/FishingDistancesDepth_byCraft"
eps.tiff(plot.cmd, flnm, width.in = 12, height.in = 6)
plot.cmd
```

#### Plotting for mesh sizes across fisheries and craft type

```
ggplot(spatial.df,aes(boat_type1, mesh1mm)) + stat_boxplot(geom ='errorbar') + geom_boxplot(notch = TRUE)  #, outlier.shape=NA)
p<-ggplot(spatial.df,aes(fishery, mesh1mm)) + stat_boxplot(geom ='errorbar') + geom_boxplot(notch = TRUE, coef = 1.5, outlier.shape=NA) + scale_y_continuous(limits = c(0, 115))
b<- p + xlab("Fishery") + ylab("Mesh size (mm)") + ggtitle("Fishery") 
c<-b + theme(axis.title.x = element_blank(), axis.title.y = element_text(face="plain", size=12))
d<-c + theme(axis.text = element_text(size=10, face="plain"))
e<-d + theme(plot.title = element_text(size = 12, face = "plain", hjust = 0.5))
e

f<-ggplot(spatial.df,aes(boat_type1,  mesh1mm)) + stat_boxplot(geom ='errorbar') + geom_boxplot(notch = TRUE, coef = 1.5, outlier.shape=NA) + scale_y_continuous(limits = c(0, 115))
f
g<- f + xlab("Type of fishing craft") + ylab("Mesh size (mm)") + ggtitle("Craft") + scale_x_discrete(breaks=c("frp", "frp_big", "kattumaram","trawl","vallam"), labels=c("Small FRP", "Big FRP", "Kattumaram", "Trawler", "Vallam"))
h<-g + theme(axis.title.x = element_blank(), axis.title.y = element_text(face="plain", size=12))
i<-h + theme(axis.text = element_text(size=10, face="plain"))
j<-i + theme(plot.title = element_text(size = 12, face = "plain", hjust = 0.5))
j

plot.cmd <- grid.arrange(e, j, layout_matrix = matrix(c(1,2), ncol=2, byrow=TRUE))
flnm <- "./Results/MeshSizesAcrossFisheriesAndCraft"
eps.tiff(plot.cmd, flnm, width.in = 12, height.in = 6)
plot.cmd
```

### Kruskal Wallis for non parametric

#### Differences in between gear, craft & regions

- Fishery and depth of fishing  

  ```
  spatial.df$fishery.f <- as.factor(spatial.df$fishery)
  kruskal.test(depth_m ~ fishery.f, data = spatial.df)
  ```

  ```
     Kruskal-Wallis rank sum test

  data:  depth_m by fishery.f
  Kruskal-Wallis chi-squared = 1111.5, df = 5, p-value < 2.2e-16
  ```
- Fishery and distance to shore  

  ```
  kruskal.test(shoredist_km ~ fishery.f, data = spatial.df)
  ```

  ```
     Kruskal-Wallis rank sum test

  data:  shoredist_km by fishery.f
  Kruskal-Wallis chi-squared = 1221, df = 5, p-value < 2.2e-16
  ```
- Fishery and mesh size  

  ```
  kruskal.test(mesh1mm ~ fishery.f, data = spatial.df)
  ```

  ```
     Kruskal-Wallis rank sum test

  data:  mesh1mm by fishery.f
  Kruskal-Wallis chi-squared = 1756.1, df = 5, p-value < 2.2e-16
  ```
- Depth and type of boat  

  ```
  spatial.df$boat_type1.f <- as.factor(spatial.df$boat_type1)
  kruskal.test(depth_m ~ boat_type1.f, data = spatial.df)
  ```

  ```
     Kruskal-Wallis rank sum test

  data:  depth_m by boat_type1.f
  Kruskal-Wallis chi-squared = 1477.9, df = 4, p-value < 2.2e-16
  ```
- Distance to shore and type of boat  

  ```
  kruskal.test(shoredist_km ~ boat_type1.f, data = spatial.df)
  ```

  ```
     Kruskal-Wallis rank sum test

  data:  shoredist_km by boat_type1.f
  Kruskal-Wallis chi-squared = 1713.8, df = 4, p-value < 2.2e-16
  ```
- Mesh size and type of boat  

  ```
  kruskal.test(mesh1mm ~ boat_type1.f, data = spatial.df)
  ```

  ```
     Kruskal-Wallis rank sum test

  data:  mesh1mm by boat_type1.f
  Kruskal-Wallis chi-squared = 953.14, df = 4, p-value < 2.2e-16
  ```
- Differences in fishing between regions  

  ```
  spatial.df$region.f <- as.factor(spatial.df$region)
  kruskal.test(depth_m ~ region.f, data = spatial.df)
  ```

  ```
     Kruskal-Wallis rank sum test

  data:  depth_m by region.f
  Kruskal-Wallis chi-squared = 300.28, df = 1, p-value < 2.2e-16
  ```
- Difference in distance to shore and regions  

  ```
  kruskal.test(shoredist_km ~ region.f, data = spatial.df)
  ```

  ```
     Kruskal-Wallis rank sum test

  data:  shoredist_km by region.f
  Kruskal-Wallis chi-squared = 193.04, df = 1, p-value < 2.2e-16
  ```
- Difference in size of mesh used and regions  

  ```
  kruskal.test(mesh1mm ~ region.f, data = spatial.df)
  ```

  ```
     Kruskal-Wallis rank sum test

  data:  mesh1mm by region.f
  Kruskal-Wallis chi-squared = 321.27, df = 1, p-value < 2.2e-16
  ```

#### post hoc with FDR correction - Benjamini-Hochberg adjustment

- By fishery  
  - Distance to shore  

    ```
    dunn.test (spatial.df$shoredist_km, g=spatial.df$fishery, method="bh", kw=TRUE, label=TRUE, 
    wrap=TRUE, table=TRUE, list=TRUE, rmc=FALSE, alpha=0.05)
    ```

    ```
      Kruskal-Wallis rank sum test

    data: x and group
    Kruskal-Wallis chi-squared = 1221.029, df = 5, p-value = 0


                   Comparison of x by group                            
                     (Benjamini-Hochberg)                              
    Col Mean-|
    Row Mean |    Gillnet       Lift       Line   Ringsein   Scoopnet
    ---------+-------------------------------------------------------
        Lift |  -1.142964
         |     0.1898
         |
        Line |  -8.697724  -0.963016
         |    0.0000*     0.2097
         |
    Ringsein |  -16.06921  -1.176209  -0.709885
         |    0.0000*     0.1996     0.2756
         |
    Scoopnet |   7.059364   2.370267   11.47283   16.32725
         |    0.0000*    0.0167*    0.0000*    0.0000*
         |
       Trawl |  -30.49773  -1.112491  -0.500257   0.491747  -19.42008
         |    0.0000*     0.1813     0.3305     0.3114    0.0000*


    List of pairwise comparisons: Z statistic (adjusted p-value)
    ------------------------------------------
    Gillnet - Lift       : -1.142964 (0.1898)
    Gillnet - Line       : -8.697724 (0.0000)*
    Lift - Line          : -0.963016 (0.2097)
    Gillnet - Ringseine  : -16.06921 (0.0000)*
    Lift - Ringseine     : -1.176209 (0.1996)
    Line - Ringseine     : -0.709885 (0.2756)
    Gillnet - Scoopnet   :  7.059364 (0.0000)*
    Lift - Scoopnet      :  2.370267 (0.0167)*
    Line - Scoopnet      :  11.47283 (0.0000)*
    Ringseine - Scoopnet :  16.32725 (0.0000)*
    Gillnet - Trawl      : -30.49773 (0.0000)*
    Lift - Trawl         : -1.112491 (0.1813)
    Line - Trawl         : -0.500257 (0.3305)
    Ringseine - Trawl    :  0.491747 (0.3114)
    Scoopnet - Trawl     : -19.42008 (0.0000)*

    alpha = 0.05
    Reject Ho if p <= alpha/2
    ```
  - Depth  

    ```
    dunn.test (spatial.df$depth_m, g=spatial.df$fishery, method="bh", kw=TRUE, label=TRUE, 
    wrap=FALSE, table=TRUE, list=TRUE, rmc=FALSE, alpha=0.05)
    ```

    ```
      Kruskal-Wallis rank sum test

    data: x and group
    Kruskal-Wallis chi-squared = 1111.5321, df = 5, p-value = 0


                   Comparison of x by group                            
                     (Benjamini-Hochberg)                              
    Col Mean-|
    Row Mean |    Gillnet       Lift       Line   Ringsein   Scoopnet
    ---------+-------------------------------------------------------
        Lift |  -0.580412
         |     0.2808
         |
        Line |  -7.436650  -1.209637
         |    0.0000*     0.1415
         |
    Ringsein |  -14.98615  -1.578500  -1.259924
         |    0.0000*     0.0954     0.1416
         |
    Scoopnet |   10.02170   2.336669   12.21471   18.01781
         |    0.0000*    0.0182*    0.0000*    0.0000*
         |
       Trawl |  -27.68484  -1.466803  -0.909434   0.836801  -21.19019
         |    0.0000*     0.1068     0.2095     0.2157    0.0000*


    List of pairwise comparisons: Z statistic (adjusted p-value)
    ------------------------------------------
    Gillnet - Lift       : -0.580412 (0.2808)
    Gillnet - Line       : -7.436650 (0.0000)*
    Lift - Line          : -1.209637 (0.1415)
    Gillnet - Ringseine  : -14.98615 (0.0000)*
    Lift - Ringseine     : -1.578500 (0.0954)
    Line - Ringseine     : -1.259924 (0.1416)
    Gillnet - Scoopnet   :  10.02170 (0.0000)*
    Lift - Scoopnet      :  2.336669 (0.0182)*
    Line - Scoopnet      :  12.21471 (0.0000)*
    Ringseine - Scoopnet :  18.01781 (0.0000)*
    Gillnet - Trawl      : -27.68484 (0.0000)*
    Lift - Trawl         : -1.466803 (0.1068)
    Line - Trawl         : -0.909434 (0.2095)
    Ringseine - Trawl    :  0.836801 (0.2157)
    Scoopnet - Trawl     : -21.19019 (0.0000)*

    alpha = 0.05
    Reject Ho if p <= alpha/2
    ```
  - Mesh size  

    ```
    dunn.test (spatial.df$mesh1mm, g=spatial.df$fishery, method="bh", kw=TRUE, label=TRUE, 
     wrap=FALSE, table=TRUE, list=TRUE, rmc=FALSE, alpha=0.05)
    ```

    ```
      Kruskal-Wallis rank sum test

    data: x and group
    Kruskal-Wallis chi-squared = 1756.0819, df = 5, p-value = 0


                   Comparison of x by group                            
                     (Benjamini-Hochberg)                              
    Col Mean-|
    Row Mean |    Gillnet       Lift       Line   Ringsein   Scoopnet
    ---------+-------------------------------------------------------
        Lift |   4.524593
         |    0.0000*
         |
        Line |   18.77161   0.075618
         |    0.0000*     0.5034
         |
    Ringsein |   3.418048  -3.996420  -14.84107
         |    0.0004*    0.0000*    0.0000*
         |
    Scoopnet |   25.21785  -0.024967  -0.348390   18.21847
         |    0.0000*     0.4900     0.4197    0.0000*
         |
       Trawl |   33.59573  -2.037622  -8.565356   13.43836  -11.12185
         |    0.0000*     0.0260    0.0000*    0.0000*    0.0000*


    List of pairwise comparisons: Z statistic (adjusted p-value)
    ------------------------------------------
    Gillnet - Lift       :  4.524593 (0.0000)*
    Gillnet - Line       :  18.77161 (0.0000)*
    Lift - Line          :  0.075618 (0.5034)
    Gillnet - Ringseine  :  3.418048 (0.0004)*
    Lift - Ringseine     : -3.996420 (0.0000)*
    Line - Ringseine     : -14.84107 (0.0000)*
    Gillnet - Scoopnet   :  25.21785 (0.0000)*
    Lift - Scoopnet      : -0.024967 (0.4900)
    Line - Scoopnet      : -0.348390 (0.4197)
    Ringseine - Scoopnet :  18.21847 (0.0000)*
    Gillnet - Trawl      :  33.59573 (0.0000)*
    Lift - Trawl         : -2.037622 (0.0260)
    Line - Trawl         : -8.565356 (0.0000)*
    Ringseine - Trawl    :  13.43836 (0.0000)*
    Scoopnet - Trawl     : -11.12185 (0.0000)*

    alpha = 0.05
    Reject Ho if p <= alpha/2
    ```
- By craft  
  - Distance to shore  

    ```
    dunn.test (spatial.df$shoredist_km, g=spatial.df$boat_type1, method="bh", kw=TRUE, label=TRUE, 
    wrap=FALSE, table=TRUE, list=TRUE, rmc=FALSE, alpha=0.05)
    ```

    ```
      Kruskal-Wallis rank sum test

    data: x and group
    Kruskal-Wallis chi-squared = 1713.7937, df = 4, p-value = 0


                   Comparison of x by group                            
                     (Benjamini-Hochberg)                              
    Col Mean-|
    Row Mean |        frp    frp_big   kattumar      trawl
    ---------+--------------------------------------------
     frp_big |  -2.102112
         |    0.0197*
         |
    kattumar |   15.37248   6.250093
         |    0.0000*    0.0000*
         |
       trawl |  -26.07743  -4.124113  -38.05761
         |    0.0000*    0.0000*    0.0000*
         |
      vallam |  -14.92838  -4.523127  -22.98987  -1.604463
         |    0.0000*    0.0000*    0.0000*     0.0543


    List of pairwise comparisons: Z statistic (adjusted p-value)
    ------------------------------------------
    frp - frp_big        : -2.102112 (0.0197)*
    frp - kattumaram     :  15.37248 (0.0000)*
    frp_big - kattumaram :  6.250093 (0.0000)*
    frp - trawl          : -26.07743 (0.0000)*
    frp_big - trawl      : -4.124113 (0.0000)*
    kattumaram - trawl   : -38.05761 (0.0000)*
    frp - vallam         : -14.92838 (0.0000)*
    frp_big - vallam     : -4.523127 (0.0000)*
    kattumaram - vallam  : -22.98987 (0.0000)*
    trawl - vallam       : -1.604463 (0.0543)

    alpha = 0.05
    Reject Ho if p <= alpha/2
    ```
  - Depth  

    ```
    dunn.test (spatial.df$depth_m, g=spatial.df$boat_type1, method="bh", kw=TRUE, label=TRUE, 
    wrap=FALSE, table=TRUE, list=TRUE, rmc=FALSE, alpha=0.05)
    ```

    ```
      Kruskal-Wallis rank sum test

    data: x and group
    Kruskal-Wallis chi-squared = 1477.8973, df = 4, p-value = 0


                   Comparison of x by group                            
                     (Benjamini-Hochberg)                              
    Col Mean-|
    Row Mean |        frp    frp_big   kattumar      trawl
    ---------+--------------------------------------------
     frp_big |  -2.157017
         |    0.0172*
         |
    kattumar |   13.59512   5.822747
         |    0.0000*    0.0000*
         |
       trawl |  -24.57939  -3.711565  -34.97901
         |    0.0000*    0.0001*    0.0000*
         |
      vallam |  -14.58273  -4.322306  -21.65827  -2.023843
         |    0.0000*    0.0000*    0.0000*    0.0215*


    List of pairwise comparisons: Z statistic (adjusted p-value)
    ------------------------------------------
    frp - frp_big        : -2.157017 (0.0172)*
    frp - kattumaram     :  13.59512 (0.0000)*
    frp_big - kattumaram :  5.822747 (0.0000)*
    frp - trawl          : -24.57939 (0.0000)*
    frp_big - trawl      : -3.711565 (0.0001)*
    kattumaram - trawl   : -34.97901 (0.0000)*
    frp - vallam         : -14.58273 (0.0000)*
    frp_big - vallam     : -4.322306 (0.0000)*
    kattumaram - vallam  : -21.65827 (0.0000)*
    trawl - vallam       : -2.023843 (0.0215)*

    alpha = 0.05
    Reject Ho if p <= alpha/2
    ```
  - Mesh size  

    ```
    dunn.test (spatial.df$mesh1mm, g=spatial.df$boat_type1, method="bh", kw=TRUE, label=TRUE, 
    wrap=FALSE, table=TRUE, list=TRUE, rmc=FALSE, alpha=0.05)
    ```

    ```
      Kruskal-Wallis rank sum test

    data: x and group
    Kruskal-Wallis chi-squared = 953.1441, df = 4, p-value = 0


                   Comparison of x by group                            
                     (Benjamini-Hochberg)                              
    Col Mean-|
    Row Mean |        frp    frp_big   kattumar      trawl
    ---------+--------------------------------------------
     frp_big |  -6.844527
         |    0.0000*
         |
    kattumar |  -6.696647   4.966940
         |    0.0000*    0.0000*
         |
       trawl |   22.50522   12.21877   26.29041
         |    0.0000*    0.0000*    0.0000*
         |
      vallam |  -2.767481   5.150123   1.108463  -14.25951
         |    0.0031*    0.0000*     0.1338    0.0000*


    List of pairwise comparisons: Z statistic (adjusted p-value)
    ------------------------------------------
    frp - frp_big        : -6.844527 (0.0000)*
    frp - kattumaram     : -6.696647 (0.0000)*
    frp_big - kattumaram :  4.966940 (0.0000)*
    frp - trawl          :  22.50522 (0.0000)*
    frp_big - trawl      :  12.21877 (0.0000)*
    kattumaram - trawl   :  26.29041 (0.0000)*
    frp - vallam         : -2.767481 (0.0031)*
    frp_big - vallam     :  5.150123 (0.0000)*
    kattumaram - vallam  :  1.108463 (0.1338)
    trawl - vallam       : -14.25951 (0.0000)*

    alpha = 0.05
    Reject Ho if p <= alpha/2
    ```
- By region  
  - Distance to shore  

    ```
    dunn.test (spatial.df$shoredist_km, g=spatial.df$region,  kw=TRUE, label=TRUE, 
    wrap=FALSE, table=TRUE, list=TRUE, rmc=FALSE, alpha=0.05)
    ```

    ```
      Kruskal-Wallis rank sum test

    data: x and group
    Kruskal-Wallis chi-squared = 193.0353, df = 1, p-value = 0


                   Comparison of x by group                            
                    (No adjustment)                                
    Col Mean-|
    Row Mean |   Pondiche
    ---------+-----------
    Tamil Na |   13.89371
         |    0.0000*


    List of pairwise comparisons: Z statistic (p-value)
    ----------------------------------------------
    Pondicherry - Tamil Nadu :  13.89371 (0.0000)*

    alpha = 0.05
    Reject Ho if p <= alpha/2
    ```
  - Depth  

    ```
    dunn.test (spatial.df$depth_m, g=spatial.df$region,  kw=TRUE, label=TRUE, 
    wrap=FALSE, table=TRUE, list=TRUE, rmc=FALSE, alpha=0.05)
    ```

    ```
      Kruskal-Wallis rank sum test

    data: x and group
    Kruskal-Wallis chi-squared = 300.2785, df = 1, p-value = 0


                   Comparison of x by group                            
                    (No adjustment)                                
    Col Mean-|
    Row Mean |   Pondiche
    ---------+-----------
    Tamil Na |   17.32854
         |    0.0000*


    List of pairwise comparisons: Z statistic (p-value)
    ----------------------------------------------
    Pondicherry - Tamil Nadu :  17.32854 (0.0000)*

    alpha = 0.05
    Reject Ho if p <= alpha/2
    ```
  - Mesh size  

    ```
    dunn.test (spatial.df$mesh1mm, g=spatial.df$region,  kw=TRUE, label=TRUE, 
    wrap=FALSE, table=TRUE, list=TRUE, rmc=FALSE, alpha=0.05)
    ```

    ```
      Kruskal-Wallis rank sum test

    data: x and group
    Kruskal-Wallis chi-squared = 321.2731, df = 1, p-value = 0


                   Comparison of x by group                            
                    (No adjustment)                                
    Col Mean-|
    Row Mean |   Pondiche
    ---------+-----------
    Tamil Na |  -17.92409
         |    0.0000*


    List of pairwise comparisons: Z statistic (p-value)
    ----------------------------------------------
    Pondicherry - Tamil Nadu : -17.92409 (0.0000)*

    alpha = 0.05
    Reject Ho if p <= alpha/2
    ```

## SPATIAL ANALYSIS

### Spatial patterns

#### Prepare the data set for neighbourhood analysis

- Create a nearest neighbour file so that each feature is assessed within the spatial context of a fixed number of its closest neighbours.  

  ```
  xy<-cbind(spatial.df$long, spatial.df$lat)
  colnames(xy) <- c("LONG","LAT")
  map_nb <-knn2nb(knearneigh(xy, longlat=TRUE))
  map_nb <-make.sym.nb(map_nb)
  map_nb
  ```

  ```
  Warning message:
  In knearneigh(xy, longlat = TRUE) : knearneigh: identical points found
  Neighbour list object:
  Number of regions: 3427 
  Number of nonzero links: 4798 
  Percentage nonzero weights: 0.04085376 
  Average number of links: 1.400058
  ```
- Create a spatial weights file  

  ```
  map_w <- nb2listw(map_nb,glist=NULL,style="W",zero.policy=FALSE)
  map_w
  ```

  ```
  Characteristics of weights list object:
  Neighbour list object:
  Number of regions: 3427 
  Number of nonzero links: 4798 
  Percentage nonzero weights: 0.04085376 
  Average number of links: 1.400058 

  Weights style: W 
  Weights constants summary:
       n       nn   S0       S1       S2
  W 3427 11744329 3427 5149.583 14944.42
  ```
- Create a non row-standardized "listw" object - for the Getis ord G analysis  

  ```
  map_b <- nb2listw(map_nb, glist=NULL, style="B", zero.policy=FALSE)
  map_b
  ```

  ```
  Characteristics of weights list object:
  Neighbour list object:
  Number of regions: 3427 
  Number of nonzero links: 4798 
  Percentage nonzero weights: 0.04085376 
  Average number of links: 1.400058 

  Weights style: B 
  Weights constants summary:
       n       nn   S0   S1    S2
  B 3427 11744329 4798 9596 31432
  ```

#### Global spatial correlation:local geary's c

- Rank boats by size/motor power  
  - Motor HP  

    ```
    gry.test(spatial.df$motor_hp1)
    ```

    | data.name | Geary C statistic | Expectation | Variance | p.value |
    | --- | --- | --- | --- | --- |
    | x |  |  |  |  |
    | weights: map\_w |  |  |  |  |
    |  | 0.226087563426783 | 1 | 0.000461813353282008 | 2.62384567961525e-284 |
  - Bottom depth  

    ```
    gry.test(spatial.df$depth_m)
    ```

    | data.name | Geary C statistic | Expectation | Variance | p.value |
    | --- | --- | --- | --- | --- |
    | x |  |  |  |  |
    | weights: map\_w |  |  |  |  |
    |  | 0.184121438849991 | 1 | 0.000668255069430153 | 6.28123697497004e-219 |
  - Distance from coast  

    ```
    gry.test(spatial.df$shoredist_km)
    ```

    | data.name | Geary C statistic | Expectation | Variance | p.value |
    | --- | --- | --- | --- | --- |
    | x |  |  |  |  |
    | weights: map\_w |  |  |  |  |
    |  | 0.00294931327704832 | 1 | 0.00082664039605247 | 8.34128907406172e-264 |
  - Boat length  

    ```
    gry.test(spatial.df$boat_length1)
    ```

    | data.name | Geary C statistic | Expectation | Variance | p.value |
    | --- | --- | --- | --- | --- |
    | x |  |  |  |  |
    | weights: map\_w |  |  |  |  |
    |  | 0.187658371162682 | 1 | 0.000489304800176196 | 1.51669871424373e-295 |
  - Total catch  

    ```
    gry.test(spatial.df$crew_size)
    ```

    | data.name | Geary C statistic | Expectation | Variance | p.value |
    | --- | --- | --- | --- | --- |
    | x |  |  |  |  |
    | weights: map\_w |  |  |  |  |
    |  | 0.344894015401117 | 1 | 0.000815596346756378 | 9.49338292882318e-117 |

### Spatial patterns for mesh size distributions

#### Prepare the data

- Create nearest neighbour and spatial weights for geary C testing spatial auto-correlation for mesh sizes  

  Subsetting the data to exclude the NA values for mesh sizes; can't run the geary's c test or getis ord because of the NAs. Create nearest neighbor file so that each feature is assessed within the spatial context of a fixed number of its closest neighbours.

  ```
  spat.df <- subset(spatial.df, !is.na(mesh1mm))
  xy<-cbind(spat.df$long, spat.df$lat)
  colnames(xy) <- c("LONG","LAT")
  plot(xy)
  map_nb <-knn2nb(knearneigh(xy, longlat=TRUE))
  map_nb <-make.sym.nb(map_nb)
  map_w <- nb2listw(map_nb, glist=NULL, style="W", zero.policy=FALSE)
  map_b <- nb2listw(map_nb, glist=NULL, style="B", zero.policy=FALSE)
  ```

  ```
  Warning message:
  In knearneigh(xy, longlat = TRUE) : knearneigh: identical points found
  ```

#### Run the Geary test

```
geary.test(spat.df$mesh1mm, map_w) # removed na.action=na.omit)
geary.test(spat.df$mesh1mm, map_w, zero.policy=NULL, 
alternative = "greater", spChk=NULL) # na.action=na.exclude, , p.adjust.method="bh"
```

```
   Geary C test under randomisation

data:  spat.df$mesh1mm 
weights: map_w 

Geary C statistic standard deviate = 23.396, p-value < 2.2e-16
alternative hypothesis: Expectation greater than statistic
sample estimates:
Geary C statistic       Expectation          Variance 
     0.4553284724      1.0000000000      0.0005420015

    Geary C test under randomisation

data:  spat.df$mesh1mm 
weights: map_w 

Geary C statistic standard deviate = 23.396, p-value < 2.2e-16
alternative hypothesis: Expectation greater than statistic
sample estimates:
Geary C statistic       Expectation          Variance 
     0.4553284724      1.0000000000      0.0005420015
```

#### Spatial autocorrelation test on categorical data:join counts

The joincount.multi function is used to tally join counts between same-colour and different colour spatial objects

- For craft type  

  ```
  spat.df$boat_type1.f <- as.factor(spat.df$boat_type1)
  jcmulti.craft <- joincount.multi(spat.df$boat_type1.f, map_w, zero.policy = FALSE, spChk = NULL, adjust.n=TRUE)
  jcmulti.craft <- as.data.frame(jcmulti.craft)
  colnames(jcmulti.craft)[4] <- "z.value"
  jcmulti.craft$p.value <- pnorm(-abs(jcmulti.craft$z.value))
  jcmulti.craft$adjusted.p.value <- p.adjust(jcmulti.craft$p.value, "fdr")
  write.csv(jcmulti.craft, file="./Results/jc_multi_craft.csv")
  jcmulti.craft
  ```

  |  | Joincount | Expected | Variance | z.value | p.value | adjusted.p.value |
  | --- | --- | --- | --- | --- | --- | --- |
  | frp:frp | 448.333333333333 | 221.529652351738 | 77.4143727087576 | 25.7774111903593 | 7.94552119506807e-147 | 2.54256678242178e-146 |
  | frp\_big:frp\_big | 4.16666666666667 | 0.184049079754601 | 0.135801478161905 | 10.8072788834754 | 1.58981043503155e-27 | 3.1796208700631e-27 |
  | kattumaram:kattumaram | 267.333333333333 | 82.4937189599766 | 40.3033663344659 | 29.1155093937937 | 1.14224765298328e-186 | 4.5689906119331e-186 |
  | trawl:trawl | 509.916666666667 | 217.233420975752 | 76.6089842455549 | 33.4393641263136 | 1.83720717837875e-245 | 1.469765742703e-244 |
  | vallam:vallam | 64.4166666666667 | 4.91849255039439 | 3.35550846525004 | 32.4806318382891 | 1.00090644424911e-231 | 5.33816770266191e-231 |
  | frp\_big:frp | 23.0416666666667 | 12.9570552147239 | 6.61538223381007 | 3.92086327961169 | 4.41161571229531e-05 | 5.42968087667115e-05 |
  | kattumaram:frp | 179.916666666667 | 270.658486707566 | 124.031469638634 | -8.14782215570812 | 1.85268372545502e-16 | 2.96429396072803e-16 |
  | kattumaram:frp\_big | 1.5 | 7.90885188431201 | 4.75839387704019 | -2.93798815597406 | 0.00165174821937064 | 0.00188771225070931 |
  | trawl:frp | 122.083333333333 | 439.100204498978 | 186.209522591467 | -23.2317235995092 | 1.08853609161107e-119 | 2.90276291096287e-119 |
  | trawl:frp\_big | 5.41666666666667 | 12.8308501314636 | 6.58016639394049 | -2.89031309527329 | 0.00192429154716521 | 0.00205257765030955 |
  | trawl:kattumaram | 33.5416666666667 | 268.022202746129 | 123.208989408158 | -21.1244592533643 | 2.37010962524273e-99 | 5.41739342912623e-99 |
  | vallam:frp | 10.9166666666667 | 66.2249488752556 | 33.0959668276582 | -9.61396715004607 | 3.49031960107625e-22 | 6.20501262413556e-22 |
  | vallam:frp\_big | 0.75 | 1.93514460999124 | 1.37288140379489 | -1.01147425810669 | 0.155894747798648 | 0.155894747798648 |
  | vallam:kattumaram | 6.25 | 40.4230207420391 | 23.603387262254 | -7.03390023473145 | 1.00419376497186e-12 | 1.46064547632271e-12 |
  | vallam:trawl | 34.4166666666667 | 65.5799006719252 | 32.911581659916 | -5.43210484651395 | 2.7846590998081e-08 | 3.71287879974413e-08 |
  | Jtot | 417.833333333333 | 1185.64066608238 | 255.932687124977 | -47.994268540164 | 0 | 0 |
- For type of net  

  ```
  spat.df$fishery.f <- as.factor(spat.df$fishery)
  jcmulti.fy <- joincount.multi(spat.df$fishery.f, map_w, zero.policy = FALSE, spChk = NULL, adjust.n=TRUE)
  jcmulti.fy <- as.data.frame(jcmulti.fy)
  colnames(jcmulti.fy)[4] <- "z.value"
  jcmulti.fy$p.value <- pnorm(-abs(jcmulti.fy$z.value))
  jcmulti.fy$adjusted.p.value <- p.adjust(jcmulti.fy$p.value, "fdr")
  write.csv(jcmulti.fy, file="./Results/jc_multi_fy.csv")
  jcmulti.fy
  ```

  |  | Joincount | Expected | Variance | z.value | p.value | adjusted.p.value |
  | --- | --- | --- | --- | --- | --- | --- |
  | Gillnet:Gillnet | 750.416666666667 | 451.184049079755 | 100.539669661172 | 29.8428437238321 | 5.43584048372764e-196 | 3.98628302140027e-195 |
  | Lift:Lift | 0 | 0.00175284837861525 | 0.00131492421018604 | -0.0483385913605902 | 0.480723199514433 | 0.480723199514433 |
  | Line:Line | 18.5833333333333 | 0.685363716038563 | 0.497070405936588 | 25.3860313969905 | 1.79877308724197e-142 | 6.59550131988724e-142 |
  | Ringseine:Ringseine | 64.4166666666667 | 6.59596844872918 | 4.42967172557744 | 27.4724606738747 | 1.87317431447735e-166 | 8.24196698370034e-166 |
  | Scoopnet:Scoopnet | 32.75 | 2.64241893076249 | 1.85076302389712 | 22.1309704005577 | 7.95580419290557e-109 | 2.50039560348461e-108 |
  | Trawl:Trawl | 491.166666666667 | 226.231375985977 | 78.2758256129964 | 29.9451148981374 | 2.54694182947884e-197 | 2.80163601242673e-196 |
  | Lift:Gillnet | 4 | 2.05433829973707 | 0.845227440513777 | 2.11631610056557 | 0.0171589661495446 | 0.0290382504069216 |
  | Line:Gillnet | 10.9166666666667 | 35.4373356704645 | 14.5301549943358 | -6.43275767288728 | 6.26546737717324e-11 | 1.5315586921979e-10 |
  | Line:Lift | 0 | 0.0806310254163015 | 0.0594444400404008 | -0.330709417787497 | 0.370431993931938 | 0.407475193325132 |
  | Ringseine:Gillnet | 30.6666666666667 | 109.393514460999 | 44.5118913994447 | -11.8000769218461 | 1.94977417202781e-32 | 5.36187897307646e-32 |
  | Ringseine:Lift | 0 | 0.248904469763365 | 0.176588500705512 | -0.592313301977417 | 0.276820405031792 | 0.338336050594413 |
  | Ringseine:Line | 2 | 4.29360210341805 | 2.99800946946988 | -1.32465132456023 | 0.0926434122381213 | 0.119891474661098 |
  | Scoopnet:Gillnet | 47.0416666666667 | 69.3339176161262 | 28.3291965521648 | -4.18829047908432 | 1.4053181308645e-05 | 2.57641657325157e-05 |
  | Scoopnet:Lift | 0 | 0.157756354075372 | 0.114295899612428 | -0.466628795609195 | 0.320382740657809 | 0.370969489182726 |
  | Scoopnet:Line | 0.416666666666667 | 2.72129710780018 | 1.93974509733649 | -1.65473694191114 | 0.0489889350189446 | 0.0718504380277855 |
  | Scoopnet:Ringseine | 4.58333333333333 | 8.40052585451359 | 5.77003040811361 | -1.58911385194109 | 0.0560173455123581 | 0.0770238500794924 |
  | Trawl:Gillnet | 172.291666666667 | 639.412795793164 | 245.846135938948 | -29.7918737356214 | 2.48910962054384e-195 | 1.36901029129911e-194 |
  | Trawl:Lift | 0 | 1.45486415425066 | 0.742551175085095 | -1.68833743001056 | 0.0456732370014522 | 0.0717722295737106 |
  | Trawl:Line | 24.75 | 25.0964066608238 | 12.6919130236038 | -0.0972350161244822 | 0.461269880469087 | 0.480723199514433 |
  | Trawl:Ringseine | 40.5833333333333 | 77.4715162138475 | 38.3785965983598 | -5.95446459712357 | 1.30462300739861e-09 | 2.60924601479723e-09 |
  | Trawl:Scoopnet | 17.4166666666667 | 49.1016652059597 | 24.5993807335103 | -6.38839269347037 | 8.38191977461669e-11 | 1.84402235041567e-10 |
  | Jtot | 354.666666666667 | 1024.65907099036 | 258.30117574792 | -41.6875802832853 | 0 | 0 |

#### Getis-Ord G-test

The Getis-Ord G statistic differs from Moran's I or Geary C by the fact that it makes a difference between high-high correlation and low-low correlation (both of which Moran's I treats as positive autocorrelation) only binary non row-standardized weight matrices work for the G-test

- Global G test  

  ```
  globalG.test(spat.df$mesh1mm, map_b)
  ```

  ```
     Getis-Ord global G statistic

  data:  spat.df$mesh1mm 
  weights: map_b 

  standard deviate = 17.778, p-value < 2.2e-16
  alternative hypothesis: greater
  sample estimates:
  Global G statistic        Expectation           Variance 
        4.893520e-04       4.092027e-04       2.032479e-11
  ```
- Local G test  

  An example to plot the localG is here: http://isites.harvard.edu/fs/docs/icb.topic923307.files/R code for Lab Ex 6.txt

  - For mesh size  

    ```
    Gmesh <- localG(spat.df$mesh1mm, map_b, zero.policy=NULL, spChk=NULL)
    summary(Gmesh)
    ```

    ```
         Min.   1st Qu.    Median      Mean   3rd Qu.      Max. 
    -2.029673 -0.720682 -0.404282  0.004912  0.756180  5.822354
    ```
  - For crew size  

    ```
    Gcrew <- localG(spat.df$crew_size, map_b)
    summary(Gcrew)
    ```

    ```
        Min.  1st Qu.   Median     Mean  3rd Qu.     Max. 
    -1.02179 -0.47916 -0.25810 -0.01024 -0.03710  8.06191
    ```

### plotting distance violations

#### Mechanised craft

```
mech <- subset(spatial.df, sector == "mech")
mechboats<-c('trawl' = "Trawler", 'vallam' = "Vallam")
sp <- ggplot(mech, aes(x=shoredist_km,y=depth_m, colour = factor (distvio))) + geom_point(shape=21, position=position_jitter(width=0.5,height=0.5))
sp
a<-sp + scale_colour_manual(name="Violation of shore distance (km)", labels=c("> 5.556km","< 5.556km"), values = c("dark grey", "black"))
b<-a + facet_grid(region ~ boat_type1, labeller = labeller(boat_type1 = as_labeller(mechboats)))
b
c<-b + ggtitle("Violation of fishing 3 nautical miles (5.556 km) from the shore by mechanized craft") + xlab("Distance from shore (km)") + ylab("Depth of substrate(m)")
d<-c + theme(strip.text.x = element_text(size=12, face="bold"), strip.text.y = element_text(size=12, face="bold"))
e<-d + theme(legend.position = "bottom")
f<-e + theme(axis.text = element_text(size=12, face="bold"))
g<-f + theme(axis.title.x = element_text(face="bold", size=14), axis.title.y = element_text(face="bold", size=14))
g
```

#### Mesh size violations

```
mesh <- ggplot(spatial.df, aes(x=shoredist_km, y=depth_m, colour = factor (meshvio))) + geom_point(shape=21, position=position_jitter(width=0.5,height=0.5)) + facet_wrap(~region)
c<-mesh + ggtitle("Violation of gear mesh sizes") + xlab("Distance from shore (km)") + ylab("Depth of substrate(m)")
d<-c + theme(strip.text.x = element_text(size=12, face="bold"), strip.text.y = element_text(size=12, face="bold"))
e<-d + theme(legend.position = "bottom")
f<-e + theme(axis.text = element_text(size=12, face="bold"))
g<-f + theme(axis.title.x = element_text(face="bold", size=14), axis.title.y = element_text(face="bold", size=14))
g + scale_colour_manual(name="Violation of gear mesh sizes(mm)", labels=c("No","Yes"), values = c("grey", "black")) + scale_size_continuous(name  ="Mesh sizes(mm)")
## creating groups to bin mesh sizes going from 0  to 120 in increments of 20.
spatial.df$mesh1mmcat<-cut(spatial.df$mesh1mm, seq(0,120,20))
## renaming the facet panels
meshcat<-c(
  '(0,20]' = "0-20",
  '(20,40]' = "20-40",
  '(40,60]' = "40-60",
  '(60,80]'= "60-80",
  '(80,100]'="80-100",
  '(100,120]'="100-120"
)
## plotting distribution of mesh sizes
p<-ggplot(spatial.df,aes(shoredist_km, depth_m, colour = factor(fishery))) +
    geom_point() +
    facet_wrap(~mesh1mmcat, scales = "free_x", labeller = labeller(mesh1mmcat = as_labeller(meshcat))) ## mesh1mmcat doesn't exit
q<- p + theme_gray() + geom_point(shape=21, position=position_jitter(width=0.5,height=0.5))
r<- q + ggtitle("Cumulative distribution of gear mesh sizes") + xlab("Distance from shore(km)") + ylab("Depth of substrate(m)")
s<- r + theme(legend.position = "bottom") + scale_colour_discrete(name  ="Fishery", 
                                                                  breaks=c("Gillnet", "Lift","Line", "Ringseine", "Scoopnet","Trawl"),
                                                                  labels=c("Gill net", "Lift net","Line", "Ring seine", "Scoop net","Trawl"))
t<-s + theme(axis.title.x = element_text(face="bold", size=14), axis.title.y = element_text(face="bold", size=14))
u<-t + theme(axis.text = element_text(size=12))
v<-u + theme(plot.title = element_text(size = 16, face = "bold")) + theme(strip.text.x = element_text(size=12, face="bold"))
x<-v + theme(legend.text = element_text(size=12)) + theme(legend.title = element_text(size=12, face = "bold"))
x
```

## Generate plots

### generate histogram of distance from shoreline

#### distance of all craft

adapted from http://www.fromthebottomoftheheap.net/2013/09/09/preparing-figures-for-plos-one-with-r/

```
ggplt <- ggplot(data=fdf, aes(fdf$shoredist_)) +
  geom_vline(xintercept = 5.556, colour="black", linetype = "longdash") +
  geom_histogram(breaks=seq(1, 45, by =0.5), 
                 ## col="gray5", # uncomment if you want grayscale
                 aes(fill=..count..)) +
  labs(x="Distance (km)", y="Count") +
  ##    theme(plot.margin = unit(c(5,4,0.05,0.05), "in")) +
  scale_fill_gradient("Count", low = "gray60", high = "black")
  ggplt
```

#### distance per craft type

```
ggplot(data=fdf, aes(fdf$shoredist_)) +
  facet_wrap (~boat_type1)+
  geom_vline(xintercept = 5.556, colour="black", linetype = "longdash") + 
  geom_histogram(breaks=seq(1, 45, by=1),
                 ## col="gray5",  # uncomment if you want grayscale
                 aes(fill=..count..)) +
  labs(x="Distance (km)", y="Count") +
  scale_fill_gradient("Count", low = "gray60", high = "black")
```

#### Depth per craft type

```
ggplot(data=fdf, aes(fdf$depth_m)) +
  facet_wrap (~boat_type1)+
  geom_histogram(breaks=seq(1, 115, by=2),
                 ## col="gray5",  # uncomment if you want grayscale
                 aes(fill=..count..)) +
  labs(x="Depth (m)", y="Count") +
  scale_fill_gradient("Count", low = "gray60", high = "black")
```

#### Processing graphs and data on violations

- Subsetting the data by district, type of craft and class of craft  
  - District  

    ```
    pon<-subset (fdf, region == "Pondicherry")
    cud<- subset (fdf,region == "Cuddalore")
    vil<- subset (fdf, region == "Villupuram")
    tn<-rbind(cud, vil)
    ```
  - Subsetting craft type: mechanised v/s artisanal for Pondy  

    ```
    trawl<-subset (pon, boat_type1 == "Trawl")
    frp<-subset(pon,boat_type1 == "FRP")
    vallam<-subset(pon,boat_type1 == "Vallam")
    kat<-subset(pon,boat_type1 == "Kattumaram")
    frpb<-subset(pon,boat_type1 == "Big FRP")
    ponart<-rbind(frp, kat, frpb)
    ponmech<-rbind(vallam,trawl)
    ```
  - Subsetting craft type: mechanised v/s artisanal for tn  

    ```
    tntrawl <- subset(tn, boat_type1 == "Trawl")
    tnfrp <- subset(tn,boat_type1 == "FRP")
    tnvallam <- subset(tn,boat_type1 == "Vallam")
    tnkat <- subset(tn,boat_type1 == "Kattumaram")
    tnfrpb <- subset(tn,boat_type1 == "Big FRP")
    tnart <- rbind(tnfrp, tnkat, tnfrpb)
    tnmech <- rbind(tnvallam,tntrawl)
    ```
- Plotting for distance violations - PONDICHERRY  

  ```
  plt <- ggplot(ponmech,aes(x=shoredist_,y=depth_m, size=crew_size, colour = factor(shoredist_>5.556))) +
   theme_gray() +
   geom_point(shape=21, position=position_jitter(width=0.5,height=0.5)) + 
   facet_wrap (~boat_type1) +
   ggtitle("MFRA Violoations of Distance of Fishing from Shore: Pondicherry") +
   xlab("Distance from shore (km)") +
   ylab("Depth of substrate(km)") +
   theme(legend.title = element_text(size = 12), plot.title = element_text(size = 12, face = "plain", hjust = 0.5)) +
   theme(legend.position="right")
  ```

  ```
  plt1.bw <- plt + scale_colour_manual(name="Violations",
    labels=c("< 5.556km","> 5.556km"), values = c("black", "darkgrey")) +
    theme(legend.title = element_text(size = 12), plot.title = element_text(size = 12, face = "plain", hjust = 0.5)) +
    scale_size_continuous(name  ="Size of crew") 
  plt1.bw
  ```

  ```
  plt1.col <- plt + scale_colour_manual(name="Violations",
                                        labels=c("< 5.556km","> 5.556km"), values = c("red", "darkgreen")) +
      theme(legend.title = element_text(size = 12)) +
      scale_size_continuous(name  ="Size of crew")
  plt1.col
  ```
- Plotting for distance violations - TAMILNADU  

  ```
  plt <- ggplot(tnmech,aes(x=shoredist_,y=depth_m, size=crew_size, colour = factor(shoredist_>5.556)))+
  geom_point(shape=1, position=position_jitter(width=0.5,height=0.5)) +
  facet_wrap (~boat_type1) +
  ggtitle("MFRA Violoations of Distance of Fishing from Shore: Tamil Nadu") +
  xlab("Distance from shore (Km)") +
  ylab("Depth of substrate(m)") +
  theme(legend.position="right", plot.title = element_text(size = 12, face = "plain", hjust = 0.5))
  ```

  ```
  plt2.col <- plt  + scale_colour_manual(name="Violations",
  labels=c("< 5.556km","> 5.556km"), values = c("red", "darkgreen")) +
  scale_size_continuous(name  ="Size of crew")
  plt2.col
  ```

  ```
  plt2.bw <- plt  + scale_colour_manual(name="Violations",
  labels=c("< 5.556km","> 5.556km"), values = c("black", "darkgrey"))+
  scale_size_continuous(name  ="Size of crew")
  plt2.bw
  ```

  ```
  mylegend <- g_legend(plt1.col)
  plot.cmd <- grid.arrange(arrangeGrob(plt1.col + theme(legend.position="none")+ xlab(""),
                        plt2.col + theme(legend.position="none"), nrow=2),
            mylegend, ncol=2, widths=c(40, 6))
  plot.cmd
  ```

  ```
   mylegend <- g_legend(plt1.bw)
   plot.cmd <- grid.arrange(arrangeGrob(plt1.bw + theme(legend.position="none")+ xlab(""),
                         plt2.bw + theme(legend.position="none"), nrow=2),
             mylegend, ncol=2, widths=c(40, 6))
  flnm <- "./Results/violations_distance_tn_pdy_bw"
  eps.tiff(plot.cmd, flnm, width.in = 9, height.in = 7.5)
  plot.cmd
  ```
- plotting for mesh violations - TAMILNADU (general 10 mm limitation)  

  ```
  tnmesh1<-subset(tn, net_type != "line")
  plt.tn <- ggplot(tnmesh1,aes(x=shoredist_,y=depth_m, size=mesh1mm, colour = factor(mesh1mm>10))) +
   geom_point(shape=1, position=position_jitter(width=0.5,height=0.5)) +
   facet_wrap (~boat_type1, nrow=1)+ggtitle("MFRA Tamil Nadu: Violations of Gear Mesh Sizes") +
   xlab("Distance from shore (Km)") +
   ylab("Depth of substrate(m)") +
   theme(legend.position="right", plot.title = element_text(size = 12, face = "plain", hjust = 0.5))
   plt.tn.col <- plt.tn +  scale_colour_manual(name="Violations",
                                     labels=c("< 10mm","> 10mm"), values = c("red", "darkgreen")) +
   scale_size_continuous(name  ="Mesh size(mm)")
   plt.tn.col
  ```

  ```
  plt.tn.bw <- plt.tn.col +  scale_colour_manual(name="Violations",
                                    labels=c("< 10mm","> 10mm"), values = c("black", "darkgrey")) +
   scale_size_continuous(name  ="Mesh size(mm)")
   plt.tn.bw
  ```
- plotting for mesh violations - PONDICHERRY (general 25 mm limitation)  

  ```
     ponmesh1 <- subset(pon, net_type != "line")
     plt.pon <- ggplot(ponmesh1,aes(x=shoredist_,y=depth_m, size=mesh1mm, colour = factor(mesh1mm>25))) +
     geom_point(shape=1, position=position_jitter(width=0.5,height=0.5)) +
      facet_wrap (~boat_type1, nrow=1)+ggtitle("MFRA Pondicherry: Violoations of Gear Mesh Sizes") +
      xlab("Distance from shore (Km)") +
      ylab("Depth of substrate(m)") +
      theme(legend.position="right", plot.title = element_text(size = 12, face = "plain", hjust = 0.5))
  plt.col.pon <- plt.pon +  scale_colour_manual(name="Violations",
                                        labels=c("< 25mm","> 25mm"), values = c("red", "darkgreen")) +
      scale_size_continuous(name  ="Mesh size(mm)")
  plt.col.pon
  ```

  ```
  plt.bw.pon <- plt.pon +  scale_colour_manual(name="Violations",
                                       labels=c("< 25mm","> 25mm"), values = c("black", "darkgrey")) +
      scale_size_continuous(name  ="Mesh size(mm)")
  plt.bw.pon
  ```

  ```
  plot.cmd <- grid.arrange(arrangeGrob(plt.bw.pon + theme(legend.position="right")+ xlab("") ,
                                       plt.tn.bw + theme(legend.position="right"), nrow=2))
  flmn <- "./Results/all_violations_mesh_tn_pdy_col"
  eps.tiff(plot.cmd, flnm, width.in = 9, height.in = 7.5)
  plot.cmd
  ```
- Subsetting the data: gillnet mesh violations - PONDICHERRY  

  ```
  pgill1 <- subset (pon, net_type == "multigill")
  pgill2 <- subset (pon,net_type == "monogill")
  pongill <- rbind(pgill1, pgill2)
  tngill1 <- subset (tn, net_type == "multigill")
  tngill2 <- subset (tn,net_type == "monogill")
  tngill <- rbind(tngill1, tngill2)
  allgill <- rbind(pongill, tngill)
  ```
- plotting for gillnet violations  

  ```
  plt <- ggplot(pongill,aes(x=shoredist_,y=depth_m, size=mesh1mm, colour = factor(mesh1mm>25))) +
   geom_point(shape=1, position=position_jitter(width=0.5,height=0.5)) + facet_wrap (~boat_type1) +
   ggtitle("Gill net mesh sizes used for fishing in Pondicherry") +
   xlab("Distance from shore (km)") +
   ylab("Depth of substrate(m)") +
   theme(legend.position="right", plot.title = element_text(size = 12, face = "plain", hjust = 0.5)) 
   plt.col <- plt + scale_colour_manual(name="Violations",
                                    labels=c("< 25mm","> 25mm"), values = c("red", "darkgreen")) +
   scale_size_continuous(name  ="Mesh size(mm)")
  plt.col # colour plot
  ```

  ```
  plt.bw <- plt + scale_colour_manual(name="Violations",
   labels=c("< 25mm","> 25mm"), values = c("darkgrey", "black")) +
   scale_size_continuous(name  ="Mesh size(mm)")
  plt.bw # grayscale plot
  ```
- Plotting for violations across districts for pondy and TN MFRA  

  ```
  plt <- ggplot(allgill,aes(x=shoredist_,y=depth_m, size=mesh1mm, colour = factor(mesh1mm>25))) +
   geom_point(aes(shape=factor(region)), position=position_jitter(width=1,height=.5))+
   labs(shape="District")+
   facet_wrap (~boat_type1) +
   ggtitle("Gill net mesh size violations: Pondicherry MFRA") +
   xlab("Distance from shore (km)") + ylab("Depth of substrate(m)") +
   theme(legend.position="right", plot.title = element_text(size = 12, face = "plain", hjust = 0.5))
  pltpdy.col <- plt + scale_colour_manual(name="Violations",
                                       labels=c("< 25mm","> 25mm"), values = c("red", "darkgreen")) +
                                       theme(legend.title = element_text(size = 12)) +
                                       scale_size_continuous(name  ="Mesh size (mm)") +
                                       scale_shape_discrete(solid=F)
   pltpdy.col # colour plot
  ```

  ```
  pltpdy.bw <- plt + scale_colour_manual(name="Violations",
                                      labels=c("< 25mm","> 25mm"), values = c("black", "darkgrey")) +
   theme(legend.title = element_text(size = 12)) +
   scale_size_continuous(name  ="Mesh size (mm)")+
   scale_shape_discrete(solid=F)
   pltpdy.bw # grayscale plot
  ```

  ```
  plt <- ggplot(allgill,aes(x=shoredist_,y=depth_m, size=mesh1mm, colour = factor(mesh1mm>10))) +
   geom_point(aes(shape=factor(region)), position=position_jitter(width=0.5,height=0.5))+
   labs(shape="District")+
   facet_wrap (~boat_type1) +
   ggtitle("Gill net mesh size violations: Tamil Nadu MFRA") +
   xlab("Distance from shore (km)") + ylab("Depth of substrate(m)") +
   theme(legend.position="right", plot.title = element_text(size = 12, face = "plain", hjust = 0.5))
   ## guides(fill=guide_legend(nrow=3,byrow=TRUE))
   plttn.col <- plt + scale_colour_manual(name="Violations",
                                      labels=c("< 10mm","> 10mm"), values = c("red", "darkgreen")) +
   scale_size_continuous(name  ="Mesh size (mm)") + 
   scale_shape_discrete(solid=F)
   plttn.col # colour plot
  ```

  ```
  plttn.bw <- plt + scale_colour_manual(name="Violations",
                                     labels=c("< 10mm","> 10mm"), values = c("black", "darkgrey")) +
   scale_size_continuous(name  ="Mesh size (mm)") +
   scale_shape_discrete(solid=F) 
   plttn.bw # grayscale plot
  ```

  - Merging the two plots  

    ```
    mylegend <- g_legend(pltpdy.col)
    pltpdy.col <- pltpdy.col + xlab("")
    grid.arrange(arrangeGrob(pltpdy.col + theme(legend.position="none")+ xlab("") ,
        plttn.col + theme(legend.position="none"), nrow=2),
        mylegend, ncol=2, widths=c(40, 6))
    ```

    ```
    mylegend <- g_legend(pltpdy.bw) 
    grid.arrange(arrangeGrob(pltpdy.bw + theme(legend.position="none")+ xlab(""),
      plttn.bw + theme(legend.position="none"), nrow=2),
      mylegend,  ncol=2, widths=c(40, 6))
    ```
- Plotting trawl net mesh violations - PONDICHERRY  

  ```
  mtrawl<-subset(ponmech, net_type == "multitrawl")
  mtrawler<-subset(ponmech, boat_type1 == "Trawl")
  ca <- ggplot(mtrawl,aes(x=shoredist_,y=depth_m, size=mesh1mm, colour = factor(mesh1mm>37))) +
   geom_point(shape=1, position=position_jitter(width=0.5,height=0.5)) +
   facet_wrap (~boat_type1)+ggtitle("Pondicherry MFRA Violations of Trawl Net Mesh Sizes") +
   xlab("Distance from shore (km)") + ylab("Depth of substrate(m)") +
   theme(legend.position="right", plot.title = element_text(size = 12, face = "plain", hjust = 0.5)) +
   scale_colour_discrete(name="Violations",labels=c("< 37mm","> 37mm")) +
   scale_size_continuous(name ="Mesh size(mm)")
  ca
  ```
- plotting for multiday fishing - PONDICHERRY  

  ```
   da <- ggplot(mtrawler,aes(x=shoredist_,y=depth_m, size=mesh1mm, colour = factor(no_days > 1))) +
    geom_point(shape=1, position=position_jitter(width=0.5,height=0.5)) +
    ggtitle("Pondicherry MFRA Violoations Multiday Trawl Fishing") +
    xlab("Distance from shore (km)") + ylab("Depth of substrate(m)") +
    theme(legend.position="right", plot.title = element_text(size = 12, face = "plain", hjust = 0.5)) +
    scale_colour_discrete(name="MFRA violation of multiday fishing",labels=c("single day fishing","multiday fishing")) +
    scale_size_continuous(name  ="Mesh sizes used")
  da # plot da
  ```
- Plotting for multiday fishing - TAMILNADU  

  ```
  ea <- ggplot(tnmech,aes(x=shoredist_,y=depth_m, size=mesh1mm, colour = factor(no_days > 1))) +
   geom_point(shape=1,  position=position_jitter(width=0.5,height=0.5)) +
   facet_wrap (~boat_type1)+ggtitle("Tamil Nadu MFRA Violoations Multiday Trawl Fishing") +
   xlab("Distance from shore (km)") + ylab("Depth of substrate(m)") +
   theme(legend.position="right", plot.title = element_text(size = 12, face = "plain", hjust = 0.5)) +
   scale_colour_discrete(name="Violations",labels=c("Single day fishing","multiday fishing")) +
   scale_size_continuous(name  ="Mesh sizes used")
   ea
  ```
- Plotting for motor HP violations - TAMILNADU  

  ```
  ij <- ggplot(tnmech,aes(x=shoredist_,y=depth_m, size=motor_hp1,colour = factor(motor_hp1>150))) +
  geom_point(shape=1,  position=position_jitter(width=1, height=1.5)) +
   facet_wrap (~boat_type1)+ggtitle("Tamil Nadu MFRA Violation of Engine Horsepower in Mechanised Craft") +
   xlab("Distance from shore (km)") + ylab("Depth of substrate(m)") +
   theme(legend.position="right", plot.title = element_text(size = 12, face = "plain", hjust = 0.5)) +
   scale_colour_discrete(name="Violations", labels=c("Mechanised <150 HP","Deep sea >150 HP"))+
   scale_size_continuous(name ="Motor HP range")
  ij
  ```
- Plotting for motor HP - PONDICHERRY  

  ```
  j <- ggplot(ponmech,aes(x=shoredist_,y=depth_m, size=motor_hp1, colour = factor(motor_hp1>150))) +
   geom_point(shape=1, position = position_jitter(width=1, height=1.5)) +
   facet_wrap (~boat_type1) +
   ggtitle("Pondicherry MFRA Violation of Engine Horsepower in Mechanised Craft") +
   xlab("Distance from shore (km)") + ylab("Depth of substrate(m)") +
   theme(legend.position="right", plot.title = element_text(size = 12, face = "plain", hjust = 0.5)) +
   scale_colour_discrete(name="Violations", labels=c("Mechanised <150 HP","Deep sea >150 HP")) +
   scale_size_continuous(name  ="Motor HP range")
  j
  ```

## Mapping

### Get the basemaps and panels

#### Stamen map

You need to be on-line for this map to work as it fetches the map from the OSM server.

```
pdymap.bw <- get_map(location = c(lon = 79.86308, lat=12.0), zoom = 9, source = "stamen", maptype = "toner-lite") 
plot(pdymap.bw)
```

#### Plot of distances

```
fdf$zone[fdf$shoredist_>5.556] <- "Non-Artisanal > 3nm (5.556km)" # changed from 3km to 3nm or 5.556km
fdf$zone[fdf$shoredist_<=5.556] <- "Artisanal <= 3nm (5.556km)"
dist.map <- qmplot(long, lat, data = fdf, maptype = "toner-lite", colour =zone, extent = "panel") +
scale_colour_manual(values = c("black", "darkgrey")) +
theme(plot.caption = element_text(colour="black", size=6, face="bold")) +
labs(x = "Longitude", y = "Latitude", colour = "Zone", caption = "NOT TO SCALE")+
scalebar(fdf,dist = 10, dd2km = TRUE, model = 'WGS84', height = 0.0075, st.size = 2.5)
plot(dist.map)
```

#### Plot panels of different variables

We use lapply to run this function quickly using the script below.

```
panels <- list("net_type", "crew_size", "boat_type1", "boat_lengt", "motor_hp1", "boat_lengt", "substrate", "mesh1mm", "catchwt_kg", "target_cat", "survey_mon", "no_days", "CraftClass","zone")
lapply(X = panels, FUN = qmplot.panels)
```

Here is an example of the output for a single map:

```
qmplot.panels("net_type")
```

## GIS data processing using GRASS and R

We use the rgrass7 library to process the GIS data. Most of the commands run functions under a version of apply to loop them over lists. We've provided some output examples where practical.

### Create a 10km grid over the data and clean up legacy files

```
execGRASS("g.mapset", mapset="craftdist") # ensure you're in the correct mapset
execGRASS("g.region", vector="BtDistUTM@PERMANENT", res="1000") # select computational extent
execGRASS("v.mkgrid", parameters = list(map="onekmgrid", box=c(1000,1000)), flags = "overwrite") # create 1km grid extending over region
execGRASS("g.remove", type="vector", name="frp,frp_big,kattumaram,trawl,vallam,frp_big_boat_type1,frp_boat_type1,kattumaram_boat_type1,trawl_boat_type1,vallam_boat_type1", flags = "f") # clean up
```

### Create subsets of maps

This calls the function `subset.map` which creates new layers based on input list by subsetting maps through v.extract

#### run for fishery

```
fishery.maps <- list("Gillnet", "Lift", "Line", "Ringseine", "Scoopnet", "Trawl") # list of maps to be created
lapply(X = fishery.maps, FUN = subset.map, colhd = "fishery") # run the function
```

#### run for boat type

```
craft.maps <- as.list(as.character(unique(fdf$boat_type1)))
lapply(X = craft.maps, FUN = subset.map, colhd = "boat_type1")
```

#### run for class of craft

```
craftclass.maps <- as.list(as.character(unique(fdf$CraftClass)))
lapply(X = craftclass.maps, FUN = subset.map, colhd = "CraftClass")
```

#### Subset map based on mesh size

This may throw an error `Column <meshsize> is already in the table. Skipping.` You can ignore this.

```
execGRASS("g.mapset", mapset="PERMANENT") #switch to PERMANENT mapset
try(execGRASS("v.db.addcolumn", map="BtDistUTM@PERMANENT", columns="meshsize INT"))#add integer column
execGRASS("v.db.update", map="BtDistUTM@PERMANENT", column="meshsize", query_column="mesh1mm") #copy values
execGRASS("g.mapset", mapset="craftdist") #switch back to craftdist mapset
lwr.mesh <- as.list(seq(0, 110, by= 20)) 
upr.mesh <- as.list(seq(20, 120, by= 20))
nm.mesh <- paste0("mesh",seq(20, 120, by=20))
execGRASS("g.remove", type = "vector", pattern = "mesh*", flags = "f", intern=TRUE) # remove existing maps HERE
mapply(FUN = subsetmesh, lwr=lwr.mesh, upr=upr.mesh, nm=nm.mesh)
```

### Process craft classes

```
CraftClass.maps <- as.list(execGRASS("g.list", type = "vector", pattern = "*CraftClass", mapset = "craftdist", intern = T))
CraftClass.facets <- gsub("_CraftClass", "", CraftClass.maps)
CraftClass.df <- do.call("rbind", mapply(read.vect.list, CraftClass.maps, CraftClass.facets, SIMPLIFY = FALSE))
qmplot.dens2d.mesh(CraftClass.df, "./Results/CraftClassMeshSizes.tiff")
qmplot.dens2d.mesh(CraftClass.df, "./Results/CraftClassMeshSizes.eps")
qmplot.dens2d.mesh(CraftClass.df, "./Results/CraftClassMeshSizes.png")
```

### Build quadrats around fisheries

```
net.maps <- as.list(execGRASS("g.list", type = "vector", pattern = "*fishery", mapset = "craftdist", intern = T))
net.map.facets <- as.list(gsub("_fishery","", net.maps))
net.df <- do.call("rbind", mapply(read.vect.list, net.maps, net.map.facets, SIMPLIFY = FALSE))
qmplot.dens2d.mesh(net.df, "./Results/NetTypesMeshSizes.tiff")
qmplot.dens2d.mesh(net.df, "./Results/NetTypesMeshSizes.eps")
qmplot.dens2d.mesh(net.df, "./Results/NetTypesMeshSizes.png")
```

### Build quadrats around mesh sizes

```
mesh.maps <- as.list(execGRASS("g.list", type = "vector", pattern = "mesh*", intern = T))
mesh.maps <- mesh.maps[c(3,4,5,6,1,2)]
mesh.map.facets <- as.list(paste(seq(0, 110, 20), "to", seq(20, 120, 20)))
mesh.df <- do.call("rbind", mapply(read.vect.list, mesh.maps, mesh.map.facets, SIMPLIFY = FALSE))
mesh.df$facet <- factor(mesh.df$facet, levels = c("0 to 20","20 to 40","40 to 60","60 to 80","80 to 100","100 to 120")) # fix order of facets
qmplot.dens2d.mesh(mesh.df, "MeshSizeClassesMeshSizes.tiff")
qmplot.dens2d.mesh(mesh.df, "MeshSizeClassesMeshSizes.eps")
qmplot.dens2d.mesh(mesh.df, "MeshSizeClassesMeshSizes.png")
```

```
boat.maps <- as.list(execGRASS("g.list", type = "vector", pattern = "*boat_type1", mapset = "craftdist", intern = T))
boat.map.facets <- as.list(gsub("_boat_type1","", boat.maps))
boats.df <- do.call("rbind", mapply(read.vect.list, boat.maps, boat.map.facets, SIMPLIFY = FALSE))
qmplot.dens2d.mesh(boats.df, "BoatTypeMeshSizes.tiff")
qmplot.dens2d.mesh(boats.df, "BoatTypeMeshSizes.eps")
qmplot.dens2d.mesh(boats.df, "BoatTypeMeshSizes.png")
```

## Generate interactive maps

### Craft and mesh size

```
p <- qmplot(x = long, y = lat, data = boats.df, maptype = "toner-lite", extent = "panel", zoom=10, geom="text", label="") +
        geom_point(data = boats.df, aes(x = long, y = lat, colour=meshsize, shape=boat_type1)) +
        stat_density2d(data = boats.df, aes(x = long, y = lat)) +
        labs(
            x = NULL,
            y = NULL ,
            title = "Craft and Mesh Sizes",
            colour = "Mesh \nSize (mm)",
            shape = "Craft Type",
            caption = NULL 
        ) +
    coord_equal()
p <- ggplotly(p)
htmlwidgets::saveWidget(p, "CraftMeshsizePlotly.html")
```

```
Coordinate system already present. Adding new coordinate system, which will replace the existing one.
```

### Mesh classes and their distribution

```
p <- qmplot(x = long, y = lat, data = mesh.df, maptype = "toner-lite", extent = "panel", zoom=10, geom="text", label="") +
        geom_point(data = mesh.df, aes(x = long, y = lat, colour=meshsize, shape=facet)) +
        stat_density2d(data = mesh.df, aes(x = long, y = lat)) +
        labs(
            x = NULL,
            y = NULL ,
            title = "Mesh Size Class and Mesh Sizes",
            colour = "Mesh \nSize (mm)",
            shape = "Mesh Size \nClass",
            caption = NULL 
        ) +
    coord_equal()
p <- ggplotly(p)
htmlwidgets::saveWidget(p, "MeshSizeClassMeshsizePlotly.html")
```

```
Coordinate system already present. Adding new coordinate system, which will replace the existing one.
```

### Net types and mesh sizes

```
p <- qmplot(x = long, y = lat, data = net.df, maptype = "toner-lite", extent = "panel", zoom=10, geom="text", label="") +
        geom_point(data = net.df, aes(x = long, y = lat, colour=meshsize, shape=facet)) +
        stat_density2d(data = net.df, aes(x = long, y = lat)) +
        labs(
            x = NULL,
            y = NULL ,
            title = "Net Types and Mesh Sizes",
            colour = "Mesh \nSize (mm)",
            shape = "Net Type",
            caption = NULL 
        ) +
    coord_equal()
p <- ggplotly(p)
htmlwidgets::saveWidget(p, "NetTypeMeshsizePlotly.html")
```

```
Coordinate system already present. Adding new coordinate system, which will replace the existing one.
```

### Craft classes and mesh sizes

```
p <- qmplot(x = long, y = lat, data = CraftClass.df, maptype = "toner-lite", extent = "panel", zoom=10, geom="text", label="") +
        geom_point(data = CraftClass.df, aes(x = long, y = lat, colour=meshsize, shape=facet)) +
        stat_density2d(data = CraftClass.df, aes(x = long, y = lat)) +
        labs(
            x = NULL,
            y = NULL ,
            title = "Class of Craft and Mesh Sizes",
            colour = "Mesh \nSize (mm)",
            shape = "Craft Class",
            caption = NULL 
        ) +
    coord_equal()
p <- ggplotly(p)
htmlwidgets::saveWidget(p, "CraftClassMeshsizePlotly.html")
```

```
Coordinate system already present. Adding new coordinate system, which will replace the existing one.
```

Author: Tara Lawrence and R.S. Bhalla

Created: 2018-03-24 Sat 19:00

Validate
