# Supplementary material for: Spatially explicit action research for coastal fisheries management
Source: PLoS One. 2018 Jul 11;13(7):e0199841. doi: 10.1371/journal.pone.0199841 (PMC6040741; doi:10.1371/journal.pone.0199841)
Supplement: S3 File — (HTML) [file pone.0199841.s005.html]

plotly
